# Growable design of passenger vehicle interior space based on FAHP and FQFD

**DOI:** 10.1371/journal.pone.0303233

**Published:** 2024-06-20

**Authors:** Zongming Liu, Xuhui Chen, Xinan Liang

**Affiliations:** 1 School of Design and Art, Shaanxi University of Science and Technology, Xi’an, Shaanxi, China; 2 School of Packaging Design and Art, Hunan University of Technology, Zhuzhou, Hunan, China; University of Campinas - UNICAMP, BRAZIL

## Abstract

The increasingly shortened development cycle of smart vehicles has led to a qualitative shift in the nature of automotive products. Growing spatial design of vehicle interiors can effectively satisfy users’ personalisation preferences and increase their willingness to buy, as well as mitigating the environmental pollution caused by the problem of rapid replacement. Considering the subjectivity and uncertainty of users’ emotional needs, this study adopts the FAHP method to comprehensively analyse and rank the SET series of factors, then combines the grey correlation method with the correlation analysis of the areas related to the interior space of the automobile, constructs the sample of the interior space of the automobile and extracts the kansei words of the space sample. Intentional vocabulary mean scores were calculated to factor analyses through kansei engineering, next the fuzzy QFD quality house was built to make affective semantic design associations and derive design weights, which are then used to guide the design and ultimately realise the design of a dynamic automotive interaction scenario. The results of the study show that the integration of different theories can reduce the uncertainties in accessing users’ emotional needs. At the same time, it can provide systematic guidance for the interaction design of a growable automobile in terms of multiple dimensions of interior space connectivity, spatial layout, and perceptual experience, as well as provide valuable suggestions for the subsequent development of interior spaces.

## 1. Introduction

At present, the artificial intelligence driving technology is gradually maturing, the intelligent transport system continues to be updated, and the life cycle of automotive products is accelerating. According to the statistics of the International Automobile Association (IAA), in 2022 a total of 85.02 million vehicles will be produced globally, which is 5 million more than in 2021. The prosperity of the automotive industry has caused irreversible damage to the environment. Xiong Yilong [1] of the Beijing University of Technology pointed out in his article that the primary energy consumption, oil consumption and greenhouse gas emissions of producing an average vehicle are 69,108 MJ, 14,545 MJ and 6,575 kg of CO2-eq. If automotive functions or equipment can grow sustainably along with human needs, it is conducive to not only help to improve the flexibility and applicability of products, and the emotional value of both parties, but also help to extend the product life cycle and reduce capacity waste due to competition between companies, and contributes to energy saving and emission reduction.

Growable Design is a design methodology that aims to create sustainable and scalable solutions. It emphasises foresight and adaptability, taking future needs and changes into account, not only to meet current needs, but also to account for future expansion and evolution. Liu Zongming [2] defined the initial concept of growable design and proposes the characteristics of growable design for product, including "DNA" hereditary, product variability, product metabolic regeneration, and user involvement. Brian Baldassarre et al. [3] integrated existing sustainable design theories and important business concepts into a framework from the perspective of business practice and summarised them into four areas: eco-design, product-service system design, sustainable business model design, and collaborative ecosystem design. Srinivasan Ananthanarayanan Bragadeesh et al. [4] proposed Harmonic Optimised Gradient Descent and Lukasiewicz Fuzzy (HOGDLF) Vehicle Lifecycle Tracking method based on Cloud Computing environment by using Blockchain and Smart Contracts for Vehicle Lifecycle Tracking, which achieved significant performance in terms of prediction time, overheads and accuracy of lifecycle. From the perspective of growth design, the external space of a vehicle is mainly reflected in its exterior design, which is the "outward appearance" of the vehicle and is part of the aesthetic experience for the user. The interior space, on the other hand, is more concerned with the system optimisation and functionality of the vehicle, which involves the design of the seating layout, the dashboard, and various interaction systems. Growth design can collect user feedback through experimentation and iterate quickly to optimise the vehicle’s interior layout and systems to enhance the user experience. Therefore, it is of great significance to apply growable design to the interior space of automobiles to meet the diversity of user needs, provide flexibility in space layout, and incorporate the frontiers of technological innovation.

## 2. Related methodology

Integrated New Product Development (iNPD) is a design methodology mainly studied by Proferssor Cagan and Vogel in their book "Creating Breakthrough Products", which is to consider user-centred design for integrated new product development [5]. The purpose of Social Economic Technological (SET) factor analysis in the iNPD methodology is to guide designers from the beginning of product development to combine artistic and scientific knowledge to continually analyse and research the three main aspects of social, economic and technological factors (collectively referred to as the SET series of factors) in order to successfully identify product opportunity gaps [6].

Fuzzy Analytic Hierarchy Process (FAHP) is a decision analysis tool that combines fuzzy logic and hierarchical analysis methods to help decision makers assign weights and prioritise through fuzzy mathematical calculations and comparisons, thus orting more accurate and targeted decision making. In order to measure the environmental and organisational performance in different designs, Hing Kai Chan et al. [7] proposed a screening model by combining Life Cycle Assessment (LCA), traditional Environmental Management Accounting (EMA) tools, and FAHP. Aungkulanon et al [8] ranked the list of barriers and sub-barriers to the adoption of electric vehicles in Thailand using the FAHP method. Moreover, the case study demonstrates that this method provides a systematic way of evaluating alternative designs, and identify options for improvement in product design. Grey Correlation Analysis (GCA) is a multi-factor analysis method based on grey theory for determining the degree of correlation between factors and the degree of influence on decision outcomes. Since this method is often used in situations of incomplete data and high uncertainty, the method can be used in conjunction with other analytical methods. Amol Nayakappa Patil et al. [9] combined FAHP and GCA and applied it to a framework for automotive choice decision making considering qualitative and quantitative criteria in order to make the best choice. Xiaoyu Li et al. [10] proposed an Incremental Capacity Analysis (ICA) method for estimating battery SOH using a combination of GCA and entropy weight methods, which was verified to be effective. Chang, XC et al [11] combined principal component analysis (PCA), gray correlation analysis (GCA), hierarchical cluster analysis (HCA) and hierarchical analysis method (AHP) for evaluating shale oil potential. Xie XJ et al [12] proposed a green design of kindergarten furniture based on the hierarchical analysis method (AHP) combined with the gray correlation analysis (GCA) Evaluation method. QFD is a quality function development methodology used to translate customer needs into specific requirements for product or service design, and QFD is often shared with other methods combining the results of quantitative and qualitative analyses for comprehensive assessment and decision making. For example, Mahmoud et al. [13] validated a proposed wheelchair design by combining the QFD framework with FAHP to determine the level of importance of engineering features. Lina He et al. [14] integrated sustainability factors into the supply chain by developing a Kano Modelling, Decision Making, Formulation, and Trial Evaluation Laboratory (DEMATEL), which was developed through a combination of non-linear programming and QFD. An interactive zero-one optimisation model combining non-linear programming and QFD was developed to analyse SC risks and select the best combination of resilience measures. Xiao J et al [15] integrated an optimization-based consensus reaching process into QFD to deal with conflicting client opinions, where client opinions were modeled on incomplete linguistic distributional evaluations. George JJ et al [16] used a Quality Function Deployment (QFD) design process for designing a rocking chair, which resulted in improved intrinsic control of the trunk in a child with a spinal cord injury (SCI).

Each analysis method has its limitations and applicability. When solving complex problems, combining multiple methods can take different factors into account and make up for the shortcomings of a single method to get more comprehensive and accurate decision-making results. FAHP is better at dealing with ambiguity and uncertainty, while grey correlation method is good at dealing with incomplete data and insufficient sample size. By combining with each other, the limitations of the various methods can be overcome and the quality of decision making can be improved. Comparison of the weights calculated using FAHP with the demand correlations derived from the QFD can help to identify potential discrepancies and contradictions.

## 3. Research framework

### 3.1 Design process

**Fig 1:**
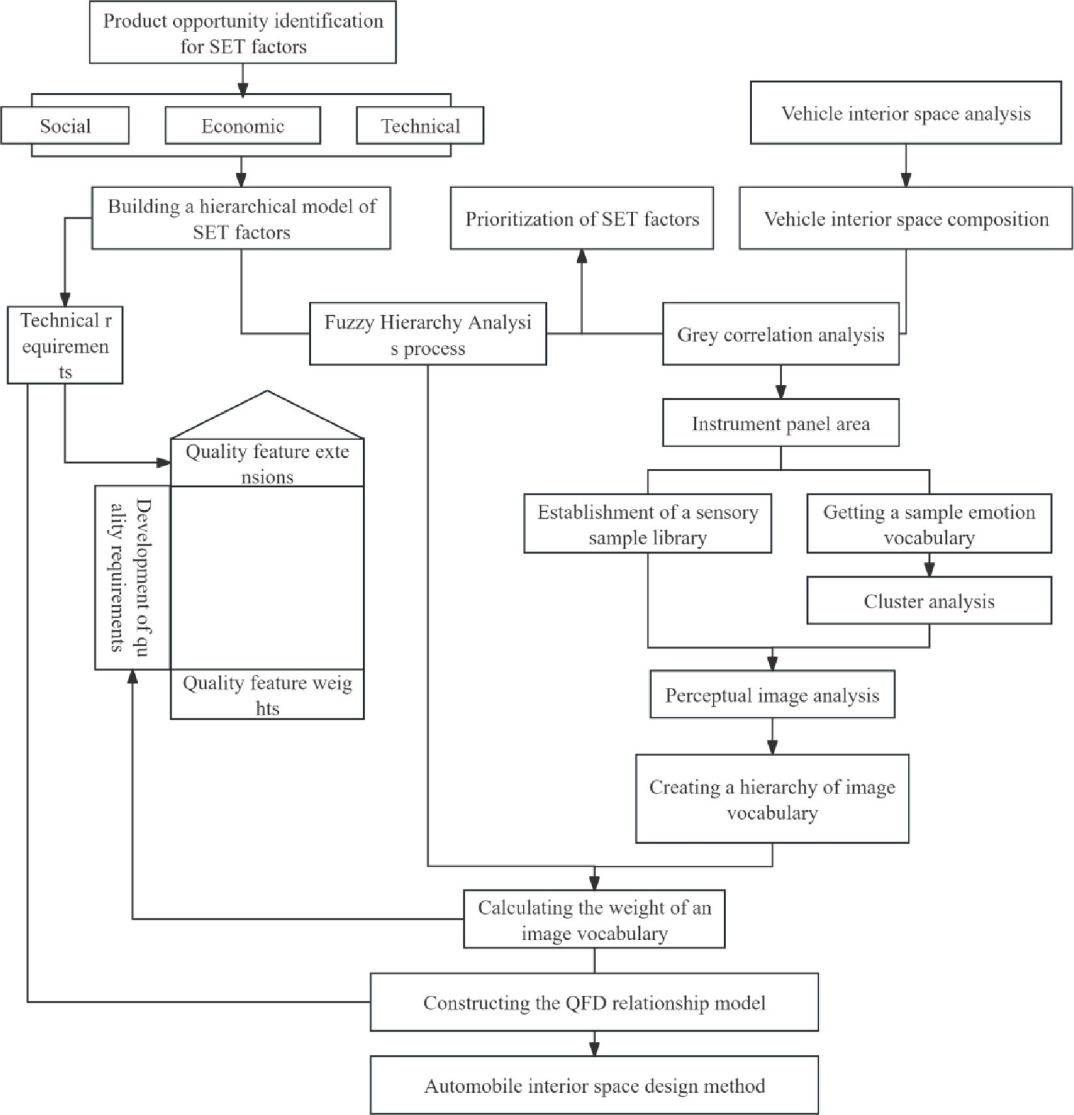
Flowchart of growable design for passenger vehicle interaction.

This study first introduces iNPD methodology to SET factor analysis of automobile interior space. SET factor hierarchical model was established by screening and selecting. FAHP method was used to calculate the comprehensive analysis and ranking of the series of factors. And the grey correlation method and the region related to automobile interior space was combined and correlation analysed. After obtaining the relevant region with the largest correlation with SET factors, the vehicle interior space sample is constructed, the kansei words of the space sample is extracted, the average score of the intentional vocabulary through the kansei engineering (KE) method is computed to vehiclery out the factor analysis, and the imagery vocabulary hierarchical model is established based on the results of the analysis. Finally, the QFD relational model is constructed by using FAHP analysis to calculate the imagery vocabulary weights and importing them into the "left wall" of the quality house, and then selecting the technical requirements corresponding to each SET factor and importing them into the "ceiling" of the quality house. Finally, the design weights are derived from the QFD and the design guidance is based on the weights. The design process is shown in Fig 1.

### 3.2 SET factor analysis of intelligent vehicle interior space

SET factor analysis is an effective tool for discovering product breakthroughs, but it can’t obtain accurate user needs. Therefore, it is necessary to obtain specific user requirements with the help of market research and use the FAHP method to calculate and rank their weights, and use the ranking results to guide the specific design.

**Fig 2:**
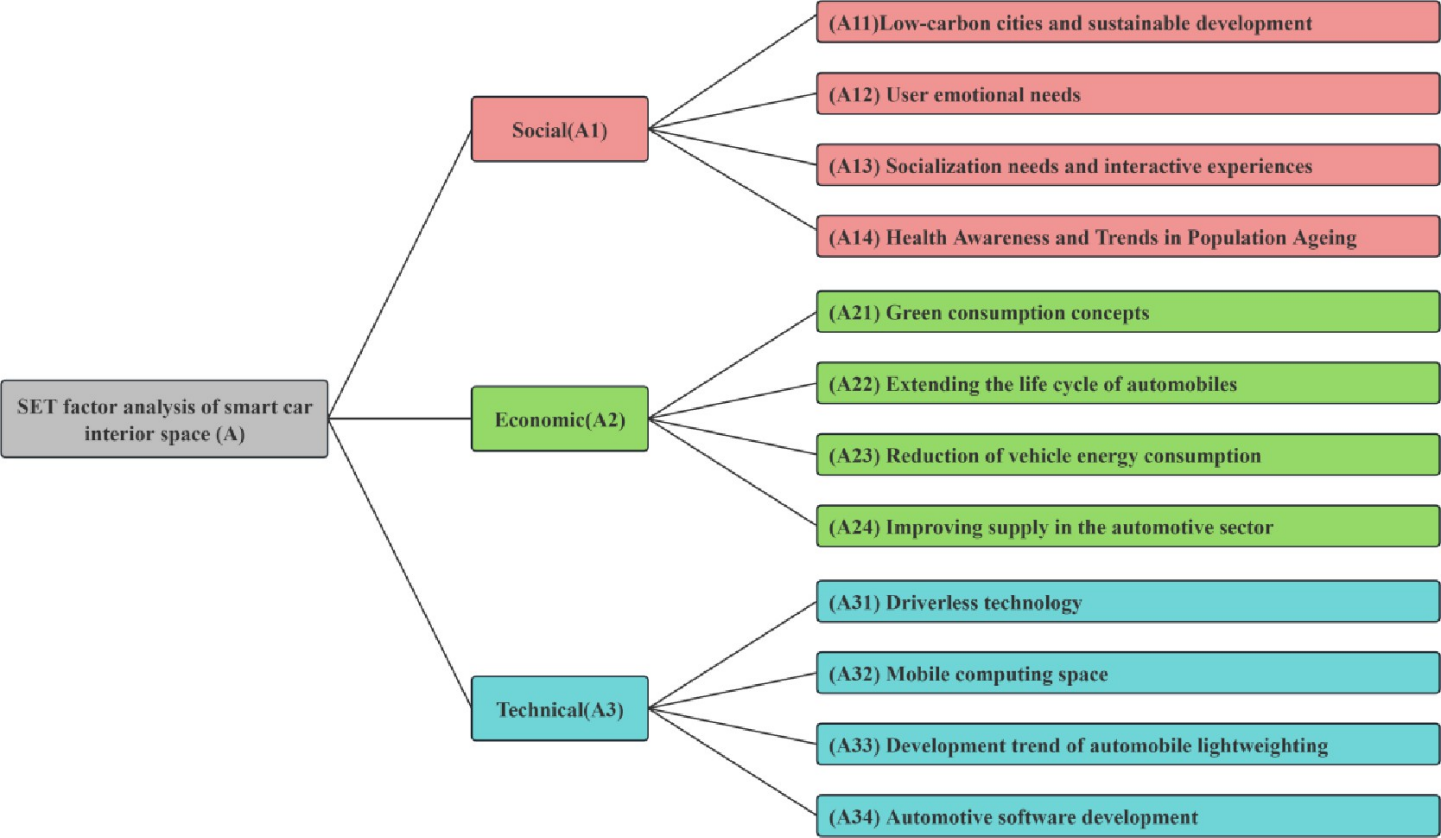
SET factors analysis for smart vehicle interior space.

Through the SET factor analysis of the interior space of the vehicle, after the discovery of product opportunity gaps, we designed a Likert scale to conduct market research on vehicle drivers and passengers, and established a hierarchical model for SET factor analysis of the vehicle, as shown in Fig 2. We used FAHP to analyse and calculate the factors, and determined the weights of the factors through fuzzy consistency test to reduce the decision-making bias, and provide direction and basis for the subsequent QFD quality function.

### 3.3 FAHP-Based SET factor prioritisation identification for vehicle interior space

FAHP is a multi-criteria decision-making method that overcomes defects such as the difficulty of the consistency test of the AHP judgment matrix and the lack of scientific basis for consistency criteria and expands the application scope of AHP. The steps for calculating the weights of each criterion and the weights of indicators corresponding to each criterion using FAHP are as follows [17].

(1)Establish a fuzzy complementary judgement matrix [18]. Through the two-by-two comparison judgement between the factors, the quantitative expression of "the relative importance of the two factors to their upper indicators (criteria)" is adopted. If the quantitative scale of 0.1~0.9 scale method shown in Table 1 is adopted, the fuzzy complementary judgement matrix *R* = (*r*_*ij*_) _*n×n*_ (*i*, *j* = 1, 2, 3,…,*n*), where *r*_*ii*_ = 0.5 means that factor *r*_*i*_ is equally important compared to itself; If rij ∈ [0.1, 0.5), it means that factor *r*_*j*_ is more important than factor *r*_*i*_; If *r*_*ij*_∈(0.5, 0.9], it means that factor *r*_*i*_ is more important than factor *r*_*j*_.

(2) Weight calculation. *R =* (*r*_*ij*_) _*n×n*_ is the fuzzy complementary judgement matrix, while W = (*W*_*1*_, *W*_*2*_, …, *W*_*i*_, …, *W*_*n*_) is the weight vector of *R*. The expression for solving the weight of the fuzzy complementary judgement matrix is as follows:

$$w_{i}=\frac{\sum_{i,j=1}^{n}r_{ij}+\frac{n}{2}-1}{n(n-1)}$$ (1)

where *W*_*i*_ is the weight of factor *r*_*i*._

(3) Consistency test. In order to judge whether the weight values calculated according to Eq (1) are reasonable, the consistency test of the comparative judgement process needs to be vehicleried out. The compatibility indexes between the judgement matrix and its feature matrix are calculated and their expressions are as follows:

$$I(A,B)=\frac{1}{n^{2}}\sum_{i,j=1}^{n}|a_{ij}+b_{ji}-1|$$ (2)

$$A={\left(a_{ij}\right)}_{n\times n}\;B={\left(b_{ij}\right)}_{n\times n}$$

$$w*={\left(w_{ij}\right)}_{n\times n}$$ (3)

$$w_{ij}=w_{i}/(w_{i}+w_{j})\;\forall i,\;j=1,\;2,\ldots ,\;n$$

Where *A*, *B* are fuzzy complementary judgement matrices. If the value of the compatibility index is less than a specific threshold *a* (generally *a* = 0.1), the judgement matrix can be regarded as a satisfactory consistency matrix. *a* is smaller, indicating that the decision maker has higher requirements for the consistency of the fuzzy judgement matrix.

**Table 1:** 0.1 To 0.9 scale method and its meaning.

Scales	Definition	Instructions
0.5	Equally important	Two factors are compared, equally important
0.6	Slightly important	Two factors are compared, one factor is slightly more important than the other
0.7	Obviously important	Two factors are compared, one factor is obviously more important than the other
0.8	Strongly important	Two factors are compared, one factor is strongly more important than the other
0.9	Extremely important	Two factors are compared, one factor is extremely more important than the other
0.1, 0.2 0.3, 0.4	Inverse comparison	If factor *r*_*i*_ is compared with factor *r*_*j*_ to obtain judgement *r*_*ij*,_ then factor *r*_*j*_ is compared with factor *r*_*i*_ to obtain judgement *r*_*j*i_ = 1-*r*_*ij*_

For the case of multiple experts involved in judging, each expert gives the fuzzy complementary judgement matrix of the same factor set according to Table 1, and the corresponding weight set can be calculated according to Eq (1), if the compatibility index between each judgement matrix and its corresponding feature matrix, as well as between any two judgement matrices is less than a specific threshold *a*, then it can be assumed that the mean value of all weight sets as the factor set’s weight assignment vector is reasonable and reliable.

According to the above steps, the SET factor weights of the interior space of the vehicle are calculated one by one, and the fuzzy consistency test is conducted to construct the fuzzy judgement matrix of the criterion layer, and the results of scoring and weight calculation are as follows:

$$A=[\begin{array}{ccc} 0.5 & 0.65 & 0.55\\ 0.35 & 0.5 & 0.4\\ 0.45 & 0.6 & 0.5 \end{array}]\;w=[\begin{array}{c} 0.3667\\ 0.2917\\ 0.3417 \end{array}]$$

Construct the fuzzy judgement matrix for each sub-criteria level and get the matrix score and weight calculation results for *A*_*1*_ as follows:

$$A_{1}=[\begin{array}{cccc} 0.5 & 0.4 & 0.55 & 0.65\\ 0.6 & 0.5 & 0.7 & 0.8\\ 0.45 & 0.3 & 0.5 & 0.6\\ 0.35 & 0.2 & 0.4 & 0.5 \end{array}]\;w_{1}=[\begin{array}{c} 0.2583\\ 0.3000\\ 0.2375\\ 0.2042 \end{array}]$$

The matrix scores and weights of *A*_*2*_ are calculated as follows:

$$A_{2}=[\begin{array}{cccc} 0.5 & 0.2 & 0.45 & 0.4\\ 0.8 & 0.5 & 0.6 & 0.55\\ 0.55 & 0.4 & 0.5 & 0.55\\ 0.6 & 0.45 & 0.45 & 0.5 \end{array}]\;w_{2}=[\begin{array}{c} 0.2125\\ 0.2875\\ 0.2500\\ 0.2500 \end{array}]$$

The matrix scores and weights of *A*_*3*_ are calculated as follows:

$$A_{3}=[\begin{array}{cccc} 0.5 & 0.6 & 0.55 & 0.3\\ 0.4 & 0.5 & 0.45 & 0.25\\ 0.45 & 0.55 & 0.5 & 0.4\\ 0.7 & 0.75 & 0.6 & 0.5 \end{array}]\;w_{3}=[\begin{array}{c} 0.2458\\ 0.2167\\ 0.2417\\ 0.2958 \end{array}]$$

The CR values of feature *A*, *A*_*1*_, *A*_*2*_, *A*_*3*_ are 0.0413, 0.0740, 0.0530, 0.0642 respectively, all of which are less than 0.1 and pass the one-time test. By multiplying the primary weight value with the secondary weight value, the comprehensive weight value of SET factors in the interior space of the vehicle can be calculated, and the comprehensive weights are sorted, see Table 2.

**Table 2:** Combined weighting of SET factors for vehicle interior space.

Factor	*A11*	*A12*	*A13*	*A14*	*A21*	*A22*	*A23*	*A24*	*A31*	*A32*	*A33*	*A34*
Combined Weight	0.0947	0.11	0.087	0.0749	0.062	0.0839	0.0729	0.0729	0.084	0.074	0.0826	0.101
Ranking	3	1	4	8	12	6	10	10	5	9	7	2

Based on the results of the calculation of the hierarchical total ranking weights of the elements, it can be seen that the most important SET factor in the social domain is the user’s emotional needs; the most important SET factor in the economic domain is the prolongation of the automobile’s life cycle; and the most important SET factor in the scientific and technological domain is the automobile’s software development.

### 3.4 Evaluation process based on GCA method

The form of vehicle interior space has important social significance what perceive the vehicle is to perceive the progress of society. The interior space of automobile has the closest contact with human beings, and its development directly depends on two basic emotional needs of human beings, speed pursuit and driving experience [19]. However, the functional emotional needs triggered by product image and preference are full of uncertainty, which is a key factor influencing users’ willing to buy. In the study of user perception, emotion and perception are essential to the study of human value judgement.

**Fig 3:**
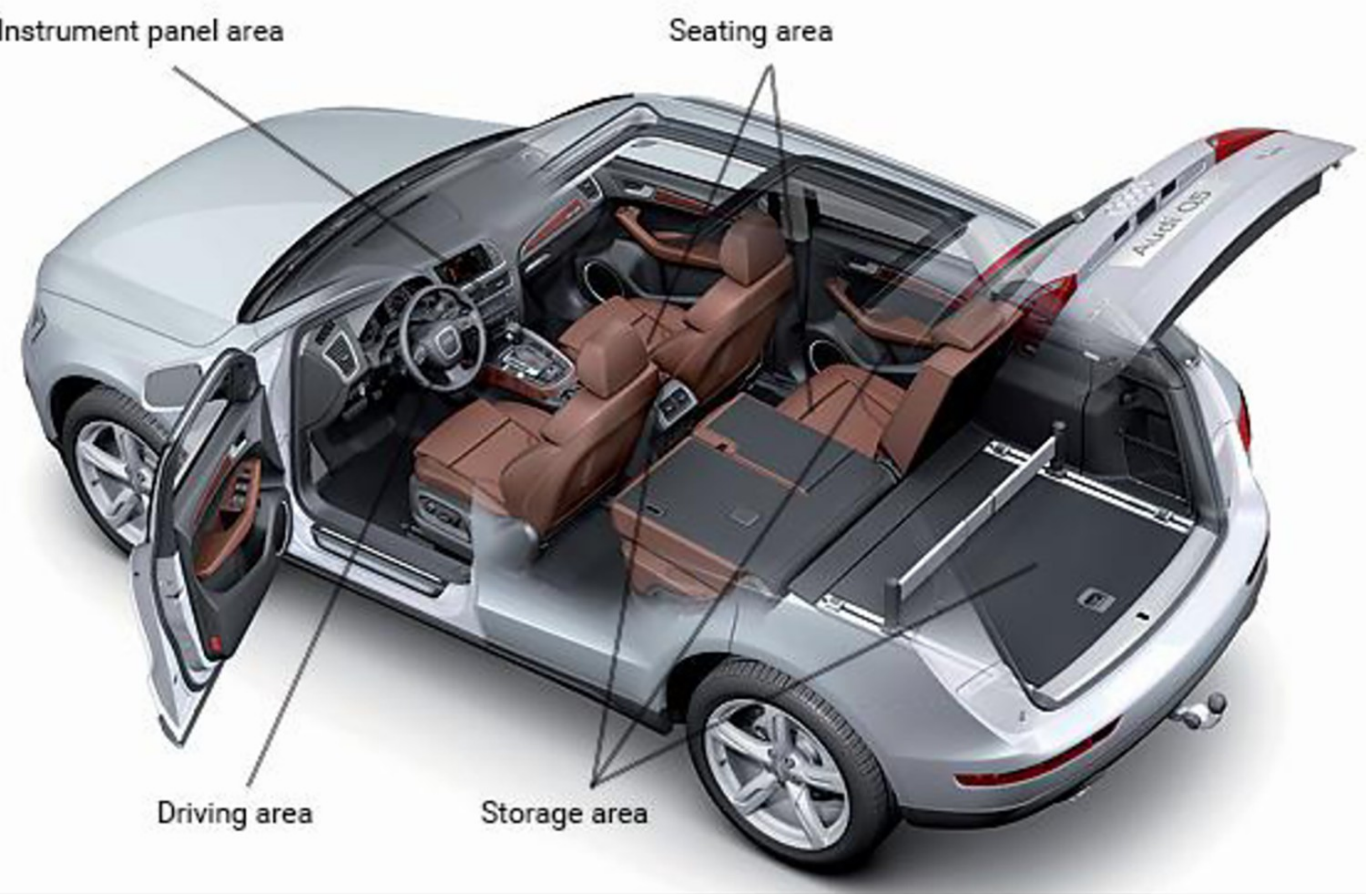
Vehicle interior space composition.

Passenger vehicle interior is the main place to achieve the function of passenger vehicle products, to a great extent, affects the user’s demand towards, but its essence in the form of the main presents a spatial characteristic, from the point of view of the composition of the interior, automotive interior including the centre console, dashboard, seats and the body interior space decoration and other parts. From a spatial point of view, it can be summarised as: dashboard area, seating area, driving area, storage area, as shown in Fig 3. The perceptual characteristics exhibited by different spaces vary. The perceptual sample library established by selecting representative areas can more accurately demonstrate the characteristics and objectives of automotive interior space design. Therefore, the grey correlation method is used to fully explore the intrinsic laws of the interior space in order to obtain representative regions.

The GCA method determines the degree of correlation between the data by comparing the geometry of the curves of the reference series and the comparison series, and the closer the geometry of the curves, the greater the degree of correlation between the data, and the smaller the opposite; the method can convert the multi-criteria decision-making problem into a single-criteria decision-making problem, and it can well balance the influence of multiple responses, like the base performance on the variables. The composition of the interior space of a vehicle is diverse and complex, the evaluation criteria of different regions are different, and the degree of correlation between each region is not clear. The grey correlation method can provide a quantitative analysis of the degree of correlation of indicators, and can complete the determination of perceptual samples in a more objective way.

The specific steps of the GCA method are generally divided into five steps [20]: establishing the evaluation matrix, determining the reference and comparison series, dimensionless processing, calculating the correlation coefficient matrix, and calculating the weighted grey correlation degree.

(1) Establishment of evaluation matrix

There are m evaluated objects and n evaluation indicators, forming the set of evaluated objects *M =* (*M*_*1*_, *M*_*2*_,…, *M*_*m*_) and the set of evaluation indicators *N* = (*N*_*1*_, *N*_*2*_,…, *N*_*n*_), and the value of *N*_*j*_ corresponding to evaluation object *M*_*i*_ is recorded as *X*_*ij*_, forming the evaluation matrix *X* as:

$$X=[\begin{array}{c} X_{1}\\ X_{2}\\ \vdots \\ X_{m} \end{array}]=[\begin{array}{cccc} x_{11} & x_{12} & \cdots & x_{1n}\\ x_{21} & x_{22} & \cdots & x_{2n}\\ \vdots & \vdots & \vdots & \vdots \\ x_{m1} & x_{m2} & \cdots & x_{mn} \end{array}]$$

(2) Determine the reference series

Take the optimal programme indicator set *X*_*0*_ *=* (*x*_01_, *x*_02_,…, *x*_0n_), where *x*_0 j_ (*j* = 1, 2,…, *n*) is the optimal value of the rank *j* indicator. When the evaluation index takes the value of the smaller the better the inverse index, *x*_0*j*_ = min(*x*_1*j*_, *x*_2*j*_, …, *x*_m*j*_); when the evaluation index takes the value of the larger the better the positive index, *x*_0*j*_ = max(*x*_1*j*_, *x*_2*j*_,…, *x*_*mj*_). From this, the reference series, i.e., the optimal set of indicators, can be determined: *X*_0_* = (*x*_01_*, *x*_02_*, …, *x*_0n_*).

(3) Determine the reference series

Take the optimal programme indicator set *X*_0_ = (*x*_01_, *x*_02_, …, *x*_0n_), where *x*_0 j_ (*j* = 1, 2, …, *n*)is the optimal value of the rank j indicator. When the evaluation index takes the value of the smaller the better the inverse index, *x*_0*j*_ = min (*x*_1*j*_, *x*_2*j*_, …, *x*_m*j*_); when the evaluation index takes the value of the larger the better the positive index, *x*_0*j*_ = max (*x*_1*j*_, *x*_2*j*_, …, *x*_*mj*_). From this, the reference series which is the optimal set of indicators can be determined: *X*_0_* = (*x*_01_*, *x*_02_*, …, *x*_0n_*).


$$U_{ij}=\frac{X_{ij}-\min X_{ij}}{\max X_{ij}-\min X_{ij}}$$ (4)

For the inverse indicator:

$$U_{ij}=\frac{\max X_{ij}-X_{ij}}{\max X_{ij}-\min X_{ij}}$$ (5)

(4) Calculate the correlation coefficient matrix

The correlation coefficient*ε*_*ij*_ of the *j* indicator of the *i* scenario with the *j* indicator of the optimal scenario is as follows at this point:

$$\varepsilon_{ij}=\frac{\min_{i}\min_{j}|{x_{0j}}^{*}-{x_{ij}}^{*}|+\lambda \max_{i}\max_{j}|{x_{0j}}^{*}-{x_{ij}}^{*}|}{\left|{x_{0j}}^{*}-{x_{ij}}^{*}|+\lambda \max_{i}\max_{j}|{x_{0j}}^{*}-{x_{ij}}^{*}\right|}$$ (6)

Where *ε*_*ij*_ is the relative difference between the *i* scheme and the optimal scheme at the *j* indicator, which describes the degree of influence of *X*_*i*_ on *X*_*0*_, and is called the correlation between *X*_*i*_ and *X*_*0*_ at the j indicator. Where *λ* is the discrimination coefficient, *λ*∈[0,1], and *λ* = 0.5 is often taken. In summary, the correlation coefficient matrix *E* can be obtained as follows:

$$E=[\begin{array}{c} E_{1}\\ E_{2}\\ \vdots \\ E_{m} \end{array}]=[\begin{array}{cccc} \varepsilon_{11} & \varepsilon_{12} & \cdots & \varepsilon_{1n}\\ \varepsilon_{21} & \varepsilon_{22} & \cdots & \varepsilon_{2n}\\ \vdots & \vdots & \vdots & \vdots \\ \varepsilon_{m1} & \varepsilon_{m2} & \cdots & \varepsilon_{mn} \end{array}]$$

(5) Calculate the weighted grey correlation

The combined weight of each sub-indicator *W* = (*w*_1_, *w*_2_, …, *w*_*n*_), satisfying *w*_*i*_ ≥ 0, $\sum_{i=1}^{n}w_{i}=1$, *R*_*i*_ (*i* = 1, 2, …, *m*) is the correlation degree of *X*_*i*_ to *X*_*0*_. According to the correlation coefficient row vector *E*_*i*_ and indicator weight vector *W* can get the following correlation degree of each programme:

$$R_{i}=E_{i}\times W=[\varepsilon_{i1},\varepsilon_{i2},\cdots ,\varepsilon_{in}]\times \left[\begin{array}{c} w_{1}\\ w_{2}\\ M\\ w_{n} \end{array}\right]$$

The correlation degree *R*_*i*_ reflects the closeness of the relationship between *X*_*i*_ and *X*_0_, so the superiority ranking of each scheme can be obtained according to the correlation degree [21].

For the evaluation of SET relevance of each area of the interior space of the vehicle on the interviews and questionnaires of 10 experts. After their comprehensive analysis of the various factors of the space areas, the four space areas were scored out of 10 respectively. Then the questionnaire data were preliminarily organised and the judging results are shown in Table 3.

**Table 3:** SET correlation evaluation for each area of vehicle interior space.

	Dashboard area	Seat area	Driving area	Storage area
*A11*	4.2	8.4	5.1	7.5
*A12*	9.3	7.9	8.6	7.4
*A13*	9.5	5.2	8.3	7.3
*A14*	6.2	7.8	7.9	6.8
*A21*	5.8	7.1	7.2	8.2
*A22*	9	7.1	7.9	5.3
*A23*	5.4	7.4	7.5	4.7
*A24*	7.7	7.6	6.2	4.4
*A31*	9.3	7.9	9.3	3.8
*A32*	7.8	9.2	8.4	8.6
*A33*	6.4	7.9	6.3	4.5
*A34*	9.5	5.8	6.6	3.9

An evaluation matrix was developed as follows:

$$X=[\begin{array}{c} X_{1}\\ X_{2}\\ X_{3}\\ X_{4} \end{array}]=[\begin{array}{cccccccccccc} 4.2 & 9.3 & 9.5 & 6.2 & 5.8 & 9.0 & 5.4 & 7.7 & 9.3 & 7.8 & 6.4 & 9.5\\ 8.4 & 7.9 & 5.2 & 7.8 & 7.1 & 7.1 & 7.4 & 7.6 & 7.9 & 9.2 & 7.9 & 5.8\\ 5.1 & 8.6 & 8.3 & 7.9 & 7.2 & 7.9 & 7.5 & 6.2 & 9.3 & 8.4 & 6.3 & 6.6\\ 7.5 & 7.4 & 7.3 & 6.8 & 8.2 & 5.3 & 4.7 & 4.4 & 3.8 & 8.6 & 4.5 & 3.9 \end{array}]$$

The above four evaluation indicators take the larger value the better positive indicator. So the reference series *X*_*0*_ = (8.4, 9.3, 9.5, 7.9, 8.2, 9.0, 7.5, 7.7, 9.3, 9.2, 7.9, 9.5) can be determined, using the method of data polarisation of the matrix *X* is normalized, according to the Formulas (4), (5) can be obtained as follows matrix X*:

$$X=[\begin{array}{c} X_{1}\\ X_{2}\\ X_{3}\\ X_{4} \end{array}]=[\begin{array}{cccccccccccc} 0 & 1 & 1 & 0 & 0 & 1 & 0.25 & 1 & 1 & 0 & 0.559 & 1\\ 1 & 0.263 & 0 & 0.941 & 0.542 & 0.487 & 0.964 & 0.97 & 0.746 & 1 & 1 & 0.339\\ 0.214 & 0.632 & 0.721 & 1 & 0.583 & 0.703 & 1 & 0.546 & 1 & 0.429 & 0.53 & 0.482\\ 0.786 & 0 & 0.488 & 0.353 & 1 & 0 & 0 & 0 & 0 & 0.572 & 0 & 0 \end{array}]$$

The correlation coefficient matrix *E* is obtained:

$$X=[\begin{array}{c} X_{1}\\ X_{2}\\ X_{3}\\ X_{4} \end{array}]=[\begin{array}{cccccccccccc} 0.333 & 1 & 1 & 0.333 & 0.333 & 1 & 0.4 & 1 & 1 & 0.333 & 0.531 & 1\\ 1 & 0.404 & 0.333 & 0.895 & 0.522 & 0.493 & 0.934 & 0.943 & 0.663 & 1 & 1 & 0.431\\ 0.389 & 0.576 & 0.642 & 1 & 0.546 & 0.627 & 1 & 0.524 & 1 & 0.467 & 0.515 & 0.491\\ 0.7 & 0.333 & 0.494 & 0.436 & 1 & 0.333 & 0.333 & 0.333 & 0.333 & 0.539 & 0.333 & 0.333 \end{array}]$$

The weighted correlation coefficient matrix as well as the composite correlation score can be obtained:

$$R=E\times W=[\begin{array}{cccccccccccc} 0.333 & 1 & 1 & 0.333 & 0.333 & 1 & 0.4 & 1 & 1 & 0.333 & 0.531 & 1\\ 1 & 0.404 & 0.333 & 0.895 & 0.522 & 0.493 & 0.934 & 0.943 & 0.663 & 1 & 1 & 0.431\\ 0.389 & 0.576 & 0.642 & 1 & 0.546 & 0.627 & 1 & 0.524 & 1 & 0.467 & 0.515 & 0.491\\ 0.7 & 0.333 & 0.494 & 0.436 & 1 & 0.333 & 0.333 & 0.333 & 0.333 & 0.539 & 0.333 & 0.333 \end{array}]\times [\begin{array}{c} 0.0947\\ 0.11\\ 0.087\\ 0.0749\\ 0.062\\ 0.0839\\ 0.0729\\ 0.0729\\ 0.084\\ 0.074\\ 0.0826\\ 0.101 \end{array}]=[\begin{array}{c} 0.059\\ 0.058\\ 0.053\\ 0.037 \end{array}]$$

In summary, R1>R2>R3>R4, so the instrument panel of the interior space of the passenger vehicle has the highest correlation with the SET factor. In the following kansei engineering calculations, the dashboard area is chosen to establish a sample pool.

### 3.5 Kansei engineering and Kansei image

Kansei engineering (KE) was first proposed in 1970 by Mitsunori Nagamachi, a professor at Hiroshima University in Japan. The theory studies and analyses users’ perceptual needs, emotions and experiences, transforms these perceptual factors into quantifiable data, and applies them to the product design and development process [22]. Kansei engineering focuses on obtaining data about users’ perceptual needs and experiences through experiments and processing and analysing the results. The design process is divided into three stages: image acquisition, model building and design optimisation [23]. In the process of using kansei engineering design methods, designers can informatise the perceptual data based on the collected kansei words through methods such as Likert scale method, SPSS statistics and semantic differential method, so as to better satisfy the perceptual needs of users [24].

Kansei image is based on Kansei engineering, which provides scientific guidance for product design and development by quantifying human perceptual elements and correlating them with elements of the target object [25]. The various stages of Kansei image research are interrelated and can generally be divided into three major stage, experimental, statistical and computer system analysis. A variety of experimental, statistical analysis and optimisation methods are involved in Kansei image, including questionnaire method, semantic differential method, factor analysis, cluster analysis, multidimensional scaling, artificial neural networks, genetic algorithms, etc [26].

**Fig 4:**
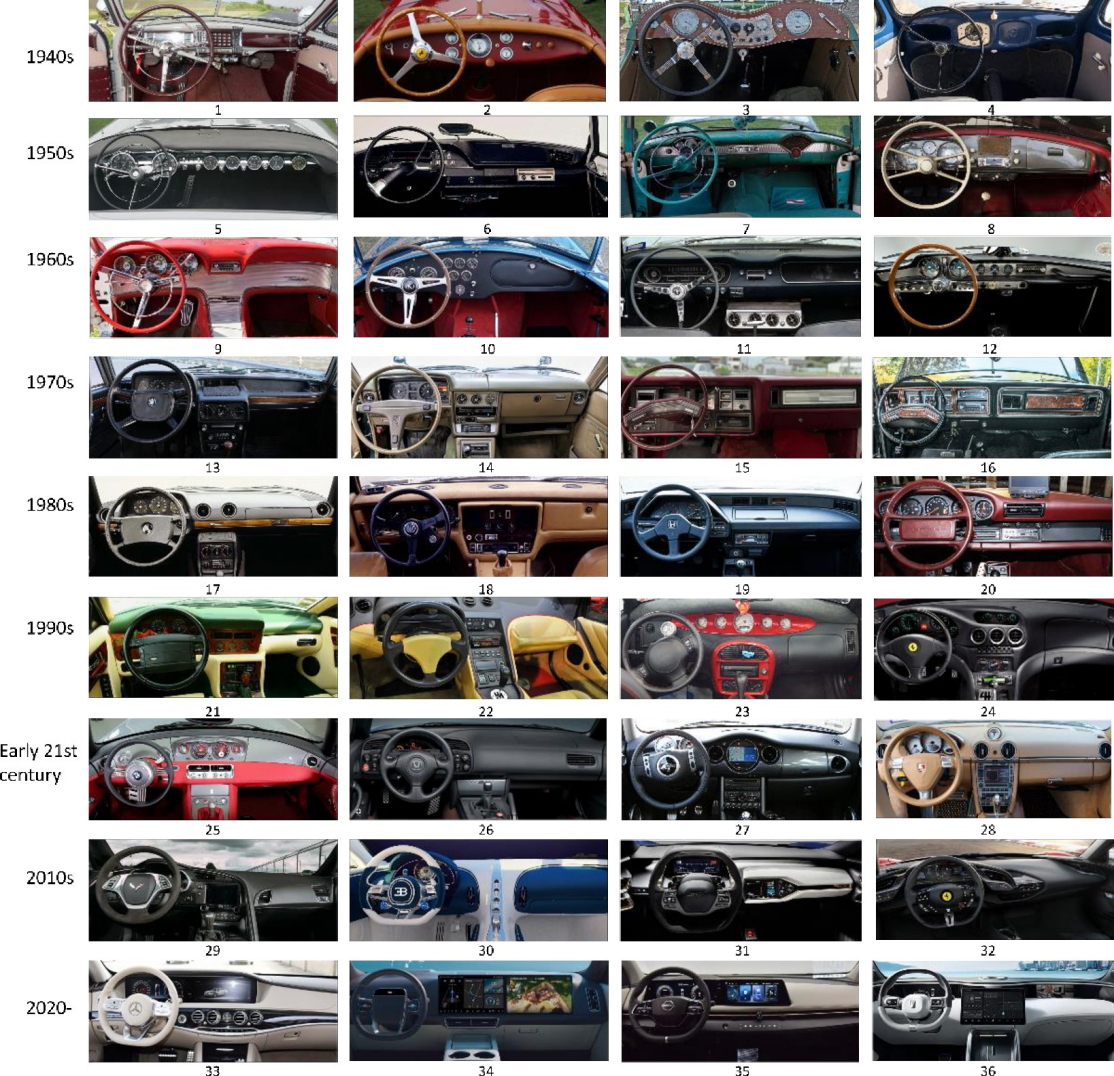
Kansei sample library.

The establishment of the sample base for the interior space of automobiles under the perspective of growable design should take into account the development trend of the interior space of automobiles, and understand the user’s expectations and needs for the interior space, and include them in the design considerations. Therefore, the selected samples should include various time periods as well as conceptual vehicle cases. According to the GCA above, it is calculated that the dashboard is most related to SET factors, and the sample library of dashboard is established as Fig 4.

Through the reading of related materials and user research, 100 Kansei image words related to the interior space of the vehicle were obtained, and 30 kansei words were initially obtained by removing the similar words. Then record the antonyms of 30 kansei words, and finally get 30 pairs of kansei word pairs. Through the questionnaire, 20 people were screened as testers and an evaluation team was set up. In order to ensure the scientificity and objectivity of the test results, taking into account the usage behaviours and cognitive characteristics of different groups, the gender ratio of the testers was basically balanced, the age distribution covered different age groups as much as possible, and the average monthly discretionary income was at a medium level, with a certain proportion of higher income. The testers were asked to record the kansei words they considered most suitable for depicting the imagery of the interior space of a vehicle against the kansei sample pool, and the kansei words is summarised in Table 4.

**Table 4:** Kansei words.

Number	Kansei Words Pairs	Number	Kansei Words Pairs
1	"Individualistic—Standardized—Avant-garde—Conservative—Progressive—Outdated	16	Spacious—crowded
2	Warm—Cold	17	Natural—artificial
3	Organized—Chaotic	18	Soft—glaring
4	Efficient—Inefficient	19	Vibrant—dull
5	Stable—Changing	20	Exclusive—shared
6	Cyclic–One-time occurrence	21	Innovative—conservative
7	Precise–Vague	22	Energy-efficient—energy-consuming
8	Comforting–Depressing	23	Practical—wasteful
9	Trendy–Out of fashion	24	Minimalist—intricate
10	Customized–Universal	25	Relaxed—tense
11	Serious–Imbalanced	26	Concise–complex
12	Intelligent–Traditional	27	Collaborative–isolated
13	Comfortable–Restrictive"	28	Fresh–murky
14	"Individualistic—Standardized—Avant-garde—Conservative—Progressive—Outdated	29	Free–restricted
15	Warm—Cold	30	Lightweight–cumbersome

#### 3.5.1 Cluster analysis of Kansei image words

Members of the evaluation team categorised the 30 pairs of kansei words pairs according to their own experience and judgement, and the number of groups was decided by the testers themselves, usually in four to six groups. The 30 pairs of kansei words in the table were listed in a 30*30 matrix, and the number of times each two pairs of kansei words were included in the same group was counted according to the categorisation results of the group, and the results were inputted into the aforementioned matrix.

**Fig 5:**
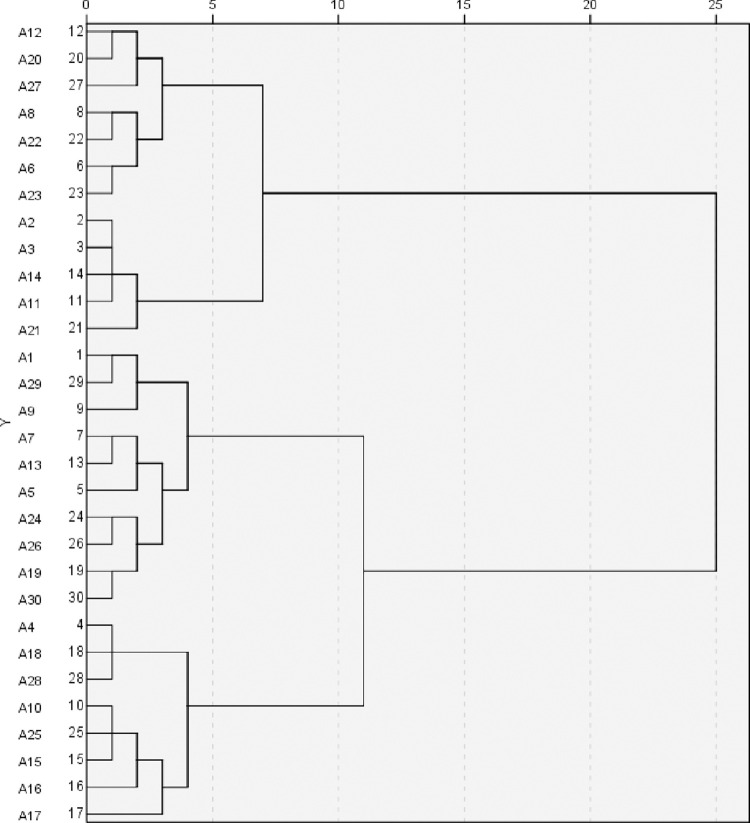
Dendrogram for cluster analysis.

According to the results of the questionnaire survey, 20 groups of 30*30 similarity coefficient matrices were averaged, and the calculated matrices were imported into the "systematic clustering" of SPSS statistical software for analysis, and the clustering method was Wald’s method, which resulted in the spectral map of kansei words as in Fig 5, and the kansei words was divided into 4 clusters with a range of 5 dotted line markers. After setting up the four clusters, the "fast clustering" analysis was used to iteratively calculate the distance from each sample to the geometric centre of the cluster, and the smaller the distance from the sample to the centre of each cluster, the more representative it is (Table 5).

**Table 5:** Kansei words to cluster centre distance.

Words pair	Distance to cluster centre	Words pair	Distance to cluster centre
6 Efficient-inefficient 8 Cyclic—Single 12 Customised—Generic 20 Proprietary—Shared 22 Energy efficient—energy consuming 23 Practical—Wasteful 27 Synergistic—isolated	21.743 21.435 19.080 18.045 22.917 18.786 23.016	1 Individualistic—Standard 2 Avant-garde—conservative 3 Advanced-Backward 11 Fashionable-Outdated 14 Intelligent—Traditional 21 Innovative—Old-fashioned	20.790 14.591 15.521 18.015 16.028 21.429
5 Orderly—chaotic 7 Stable—changing 9 Precise—Ambiguous 13 Steady—Imbalanced 19 Spiritual—Dull 24 Simple—Complex 26 Simple—Complex 29 Free—Restricted 30 Lightweight—bulky	24.339 22.285 24.130 23.536 22.441 19.421 19.071 22.510 24.066	4 Warm—Cold 10 Soothing—Depressing 15 Cosy—Restricted 16 Spacious—Crowded 17 Natural—Artificial 18 Soft—harsh 25 Relaxing—Tight 28 Fresh—Turbid	19.155 20.265 21.608 24.823 27.091 23.694 19.369 23.461

By cluster analysis of the data obtained from the research, four sets of clusters were finally compiled, as shown in Table 6. Through the distance from the samples to the geometric centre of the cluster, 3 representative word pairs with the smallest distance were selected from each group, the first group being: 12 customised—generic, 20 exclusive—shared, 23 practical—wasteful; the second group being: 2 avant-garde—conservative, 3 advanced—backward, 14 intelligent—traditional, the third group being: 7 stable—changing, 24 simple—complicated, 26 simple-complex, and the fourth group is: 4 warm-cold, 10 soothing-depressing, 25 relaxed-tight.

**Table 6:** Kansei words clustering.

Group 1	Group 2
6 Efficient—inefficient 8 Cyclic—Single 12 Customised—Generic 20 Proprietary—Shared 22 Energy efficient—energy consuming 23 Practical—Wasteful 27 Synergistic—isolated	1 Individualised—Standard 2 Avant-garde—conservative 3 Advanced—Backward 11 Fresh—Outdated 14 Intelligent—Traditional 21 Innovative—Old-fashioned
Group 3	Group 4
5 Orderly—Chaotic 7 Stable—Changing 9 Precise—Ambiguous 13 Stable—Imbalanced 19 Spiritual—Stagnant 24 Simple—Complex 26 Simple—Complex 29 Free—Restricted 30 Lightweight—bulky	4 Warm—Cold 10 Soothing—Depressing 15 Cosy—Restricted 16 Spacious—Crowded 17 Natural—Artificial 18 Soft—harsh 25 Relaxing—Tight 28 Fresh—Turbid

#### 3.5.2 Analysis of perceptual measures of vehicle interior space

The perceptual evaluation of the dashboard samples was vehicleried out through questionnaires and a semantic differential 5 order scale was used to collect data. The range of perceptual values is -2, -1, 0, 1, 2 to form the semantic differential score of the vehicle dashboard, 53 questionnaires were collected, 50 valid questionnaires were obtained after excluding invalid questionnaires, and the mean value of the score of the kansei words of each sample was calculated, which is shown in Table 7.

**Table 7:** Mean Kansei words score.

number	2	3	4	7	10	12	14	20	23	24	25	26
sample 1	1.31	1.25	1.69	1.77	0.85	-1.23	-1.82	0.41	1.54	-0.87	-0.58	-0.79
sample 2	1.64	1.54	1.78	0.24	1.87	-1.33	-1.74	0.22	1.74	1.85	1.56	1.76
sample 3	1.69	1.34	1.66	-0.22	1.72	-1.41	-1.53	0.54	1.48	-0.24	1.49	-0.18
sample 4	1.55	1.25	-1.21	1.42	0.24	-1.35	-1.48	0.64	1.54	1.54	1.28	1.48
sample5	1.84	1.68	-1.88	1.71	0.14	-1.24	-1.38	0.27	1.47	1.48	-1.48	1.51
sample6	1.78	1.58	-1.95	1.74	-0.42	-1.54	-0.47	0.47	1.24	1.64	-1.37	1.61
sample7	1.74	1.71	-0.85	0.54	0.75	-1.48	-1.35	0.67	1.57	1.72	0.57	1.8
sample8	1.34	1.05	1.84	-0.41	1.24	-1.24	-1.48	0.21	1.24	1.61	1.04	1.48
sample9	1.68	1.62	1.92	-0.21	1.75	-0.88	-1.24	0.05	1.47	1.87	1.74	1.77
sample10	1.36	1.24	-0.85	-0.1	0.86	-1.47	-0.97	0.35	1.25	-0.41	-0.21	-0.28
sample11	1.79	1.35	-0.95	0.24	0.14	-1.53	-0.85	0.84	1.52	1.71	-0.73	1.62
sample12	1.68	1.54	-0.81	0.84	0.43	-1.48	-1.54	0.27	1.42	0.18	-1.49	0.22
sample13	1.35	1.2	-0.51	1.24	0.45	-1.54	-0.46	0.59	1.45	0.94	-0.82	0.82
sample14	-0.6	1.24	1.05	1.48	1.05	-1.68	-1.17	0.42	1.64	-0.46	-1.04	-0.35
sample15	1.56	1.3	0.86	1.74	-0.48	-1.58	-1.05	0.28	1.74	0.72	-1.26	0.69
sample16	1.35	1.51	-0.74	1.72	-0.21	-1.37	-1.32	0.74	1.25	0.28	-0.58	0.17
sample17	-0.87	1.34	-0.81	1.84	0.83	-1.56	-0.51	0.68	1.45	-0.28	1.04	-0.11
sample18	1.65	1.72	0.25	0.84	-1.42	-1.17	-0.41	0.48	1.47	1.08	-0.84	1.24
sample19	0.36	1.58	-1.52	0.72	-0.97	-1.55	-1.32	0.42	1.58	0.84	-1.22	0.22
sample20	1.25	1.33	1.49	0.18	-0.85	-1.05	-0.42	0.57	1.85	0.17	-0.24	0.25
sample21	1.28	1.05	1.65	1.26	0.14	-1.44	-1.42	0.51	1.57	-0.16	0.57	-0.02
sample22	0.39	1.62	1.84	1.74	1.05	-1.74	-0.38	0.38	1.9	-0.72	-0.17	-0.88
sample23	0.62	1.21	0.51	0.17	1.62	-1.49	-1.42	0.37	1.64	-0.57	0.94	-0.42
sample24	0.15	1.64	-1.94	1.86	0.21	-1.56	-0.25	0.52	1.35	-0.17	-0.28	0.22
sample25	-1.22	1.05	0.44	0.87	1.51	-1.52	-0.21	0.84	1.58	1.15	1.24	1.05
sample26	0.95	1.76	-1.75	1.02	-1.32	-1.28	-1.32	0.49	1.26	1.72	-0.48	1.79
sample27	-1.11	1.82	-0.62	1.81	0.15	-1.02	-0.28	0.38	1.48	-1.04	0.26	-0.74
sample28	-1.05	1.68	1.57	1.73	-0.51	-1.2	-0.14	0.43	1.57	-1.24	-0.68	-1.35
sample29	0.85	1.62	-1.69	0.98	-0.25	-1.18	-0.19	0.28	1.48	-0.58	-0.15	-0.25
sample30	1.72	1.84	-0.54	-0.71	0.15	0.48	0.41	0.64	1.16	1.81	1.24	1.75
sample31	0.95	1.22	-0.95	0.17	-0.81	-0.97	-0.18	0.21	1.28	1.21	-0.99	1.36
sample32	0.21	1.01	-1.72	0.84	-0.77	-1.21	-0.27	0.69	1.65	0.95	-1.26	1.24
sample33	1.58	1.84	0.25	1.24	1.54	0.55	0.84	0.07	1.58	1.84	0.82	1.64
sample34	1.05	1.72	-1.05	-0.41	1.62	1.05	1.28	-1.05	1.48	1.72	-0.51	1.84
sample35	1.61	1.71	0.01	1.04	1.71	0.94	0.94	-0.08	1.47	1.81	0.06	1.74
sample36	1.23	1.62	-0.24	0.42	1.54	1.61	1.18	-0.15	1.38	1.69	0.18	1.68

The mean values of kansei words were imported into the SPSS data analysis software for the downscaling of the data. Table 8 shows the variance of the common factor, which shows the tendency of each sample of kansei words, and the closer the cumulative contribution of the variance of the common factor is to 0.9 kansei words is more likely to influence the user’s judgement.

**Table 8:** Common factor variance.

	Start	Capture
2 Avant-garde—conservative	1.000	0.570
3 Advanced—Backward	1.000	0.461
4 Warm—Cold	1.000	0.711
7 Stable-Changing	1.000	0.563
10 Soothing—Depressing	1.000	0.713
12 Customised—Generic	1.000	0.886
14 Intelligent—Traditional	1.000	0.776
20 Exclusive—Shared	1.000	0.625
23 Practical—Wasteful	1.000	0.347
24 Simple—Complicated	1.000	0.840
25 Relaxed—Tight	1.000	0.611
26 Simple—Complex	1.000	0.859

**Fig 6:**
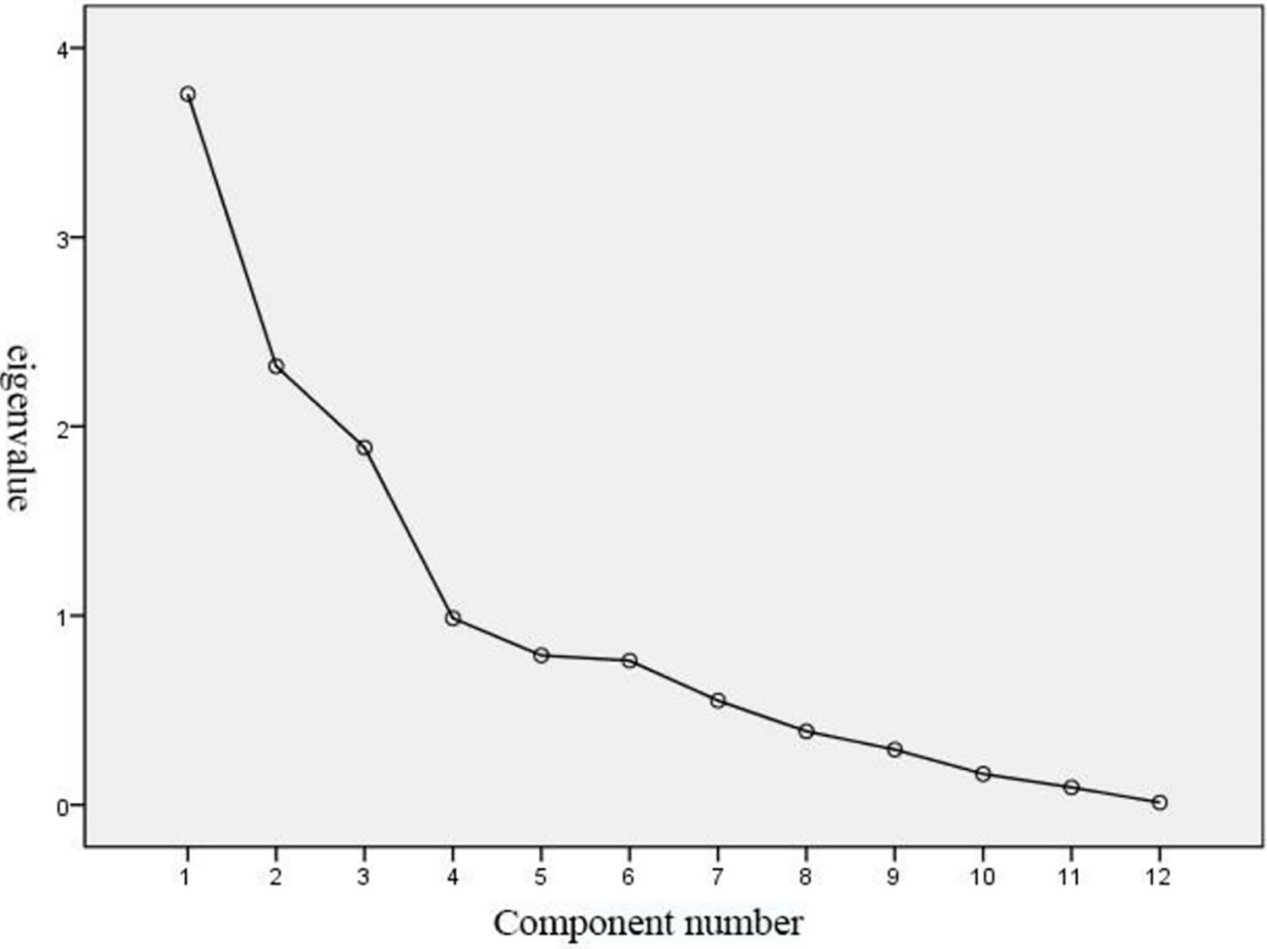
Factor eigenvalue steep slope diagram.

SPSS software factor analysis of statistical data, through the factor eigenvalue steep slope diagram (see Fig 6) can be seen that there are three indicators greater than 1. Therefore, only need to retain the first three factors can summarise most of the information of the statistical data, according to the eigenvalue to determine the extraction of the first, second and third principal components.

Kaiser normalised maximum variance is a commonly used orthogonal rotation method for factor analysis, which aims to make the rotated factor loading matrix easier to interpret and decipher. Orthogonal rotation using Kaiser’s Normalised Maximum Variance method reduces the correlation of the perceptual factors across factors and the rotated factor loading matrix is shown in Table 9.

**Table 9:** Rotated factor loading matrix.

Kansei Image	Ingredients		
	1	2	3
2 Avant-garde—conservative	0.750	-0.069	0.050
3 Advanced—Backward	0.084	0.627	-0.247
4 Warm—Cold	-0.258	-0.168	0.785
7 Stable-Changing	-0.581	-0.087	-0.467
10 Soothing—Depressing	0.042	0.250	0.805
12 Customised—Generic	0.303	0.872	0.183
14 Intelligent—Traditional	-0.016	0.877	-0.077
20 Exclusive—Shared	-0.127	-0.742	-0.241
23 Practical—Wasteful	-0.459	-0.123	0.349
24 Simple—Complicated	0.890	0.220	-0.005
25 Relaxed—Tight	0.169	-0.019	0.763
26 Simple—Complex	0.895	0.237	-0.032

According to the rotated factor loading matrix, the perceptual words that have a strong relationship with each principal component can be analysed, and the larger the absolute value indicates the strongest relationship with the principal component. Excluding pairs of words with similar meanings, it can be seen that the kansei words closely related to principal component 1 are avant-garde-conservative, stable-varying, and simple-complicated; the kansei words closely related to principal component 2 are customised-universal, intelligent-traditional, and exclusive-shared; and the perceptual words closely related to principal component 3 are warm-cold, soothing-oppressive, and relax-tight.

### 3.6 FAHP analysis of vehicle interior space Kansei image

**Fig 7:**
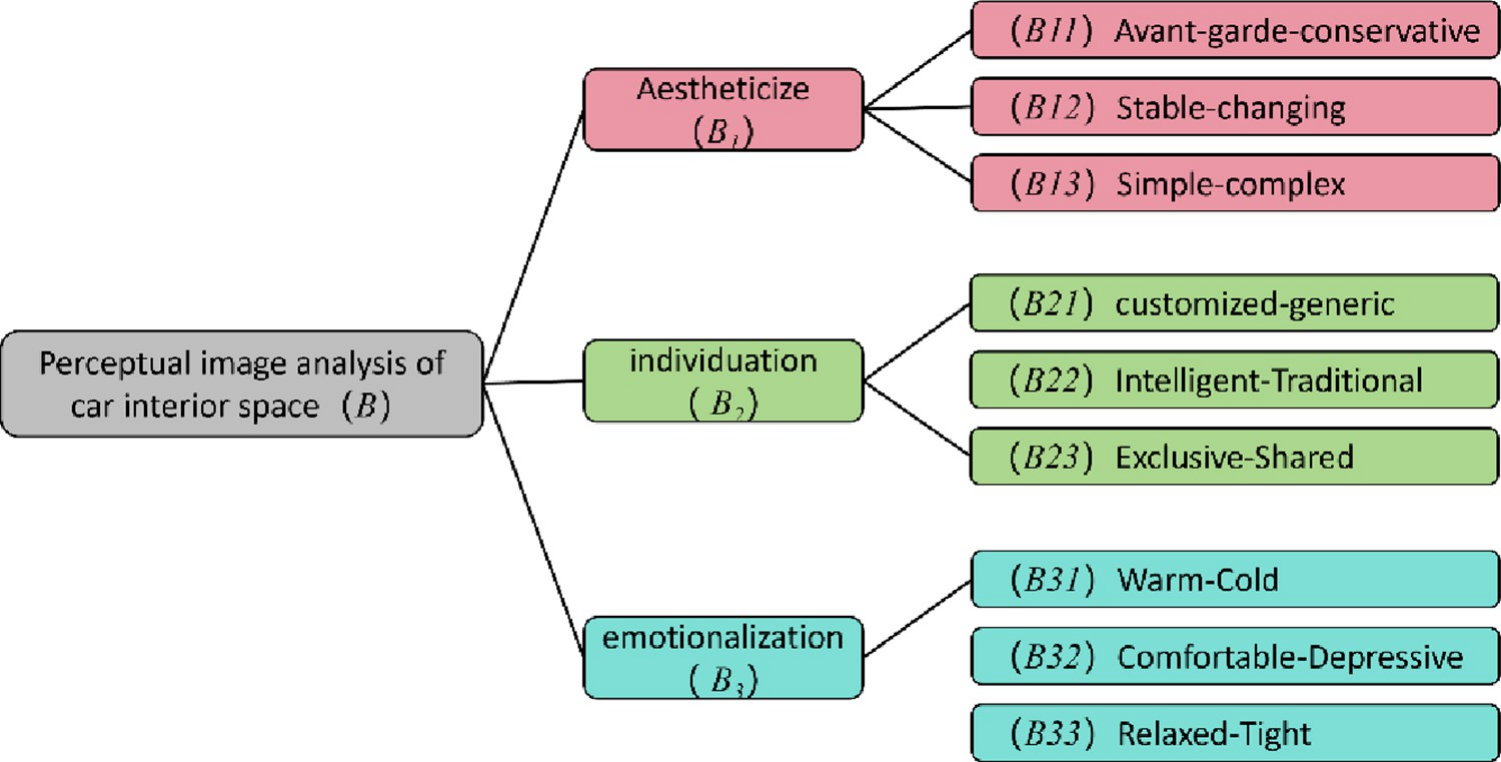
Hierarchical model.

Through the results of the vehicle interior space imagery study above, combined with the characteristics of vehicle interior space design, a group discussion was conducted, with a total of 10 group members including design department teachers, graduate students and automotive industry related personnel. The sub-criteria layer selects 9 pairs of kansei words pairs in the rotated factor loading matrix that have a close relationship with the principal components, and combined with the results of the factor analysis of kansei words, the final selected criterion layer is: aesthetics, personalisation, and emotionality. The final vehicle interior space kansei image hierarchy model is shown in Fig 7.

According to the above steps, use FAHP to calculate the kanse image weights of the interior space of the vehicle one by one, and vehiclery out fuzzy consistency test to construct the fuzzy judgement matrix of the criterion layer, and the results of the scoring and weight calculation are as follows:

$$B=[\begin{array}{ccc} 0.5 & 0.27 & 0.35\\ 0.73 & 0.5 & 0.55\\ 0.65 & 0.45 & 0.5 \end{array}]\;w=[\begin{array}{c} 0.27\\ 0.38\\ 0.35 \end{array}]$$

Construct the fuzzy judgement matrix of each sub-criteria level and get the matrix score and weight calculation results of B1 as follows:

$$B_{1}=[\begin{array}{ccc} 0.5 & 0.72 & 0.36\\ 0.28 & 0.5 & 0.25\\ 0.64 & 0.75 & 0.5 \end{array}]\;w=[\begin{array}{c} 0.3467\\ 0.255\\ 0.3983 \end{array}]$$

The matrix score and weight calculation results of *B*_*2*_ are as follows:

$$B_{2}=[\begin{array}{ccc} 0.5 & 0.36 & 0.53\\ 0.64 & 0.5 & 0.66\\ 0.47 & 0.34 & 0.5 \end{array}]\;w=[\begin{array}{c} 0.315\\ 0.3833\\ 0.3017 \end{array}]$$

The results of matrix scoring and weight calculation for *B*_*3*_ are as follows:

$$B_{3}=[\begin{array}{ccc} 0.5 & 0.26 & 0.34\\ 0.74 & 0.5 & 0.54\\ 0.66 & 0.46 & 0.5 \end{array}]\;w=[\begin{array}{c} 0.2667\\ 0.38\\ 0.3533 \end{array}]$$

The CR values of feature *B*, *B*_*1*_, *B*_*2*_, *B*_*3*_ are 0.0579, 0.0866, 0.0468, and 0.0588 respectively, which are less than 0.1 and pass the one-time test. By multiplying the first-level weight value with the second-level weight value, the comprehensive weight value of the sensual imagery of the interior space of the vehicle can be calculated, and the comprehensive weights are ranked, as shown in Table 10.

**Table 10:** Comprehensive weights of Kansei image in vehicle interior space.

Factor	*B11*	*B12*	*B13*	*B21*	*B22*	*B23*	*B31*	*B32*	*B33*
Combined Weighting	0.0936	0.0689	0.1075	0.1197	0.1457	0.1146	0.0933	0.1330	0.1237
Ranking	7	9	6	4	1	5	8	2	3

### 3.7 Quality house development

**Fig 8:**
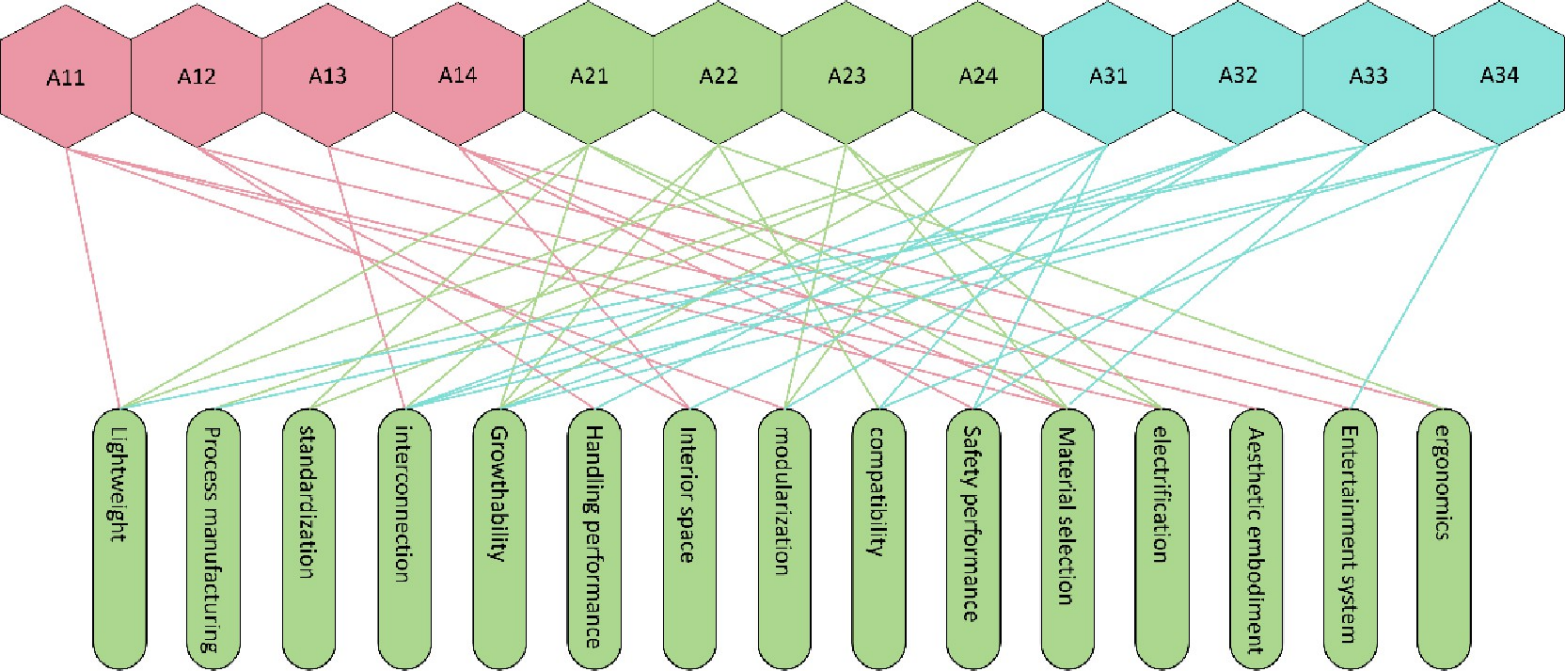
Correspondence between SET factors and technical requirements.

The weights of the kansei image of the interior space have been calculated using FAHP, and the kansei words pairs and their weights are directly imported into the "left wall" of the quality house, and the next step is to determine the technical requirements located in the "ceiling". This involves the transformation of requirements and technology, through the analysis and refinement of SET factors, 11 technical requirements are obtained, of which the correspondence between SET factors and technical requirements is shown in Fig 8. The "roof" of the quality house is the correlation analysis between the technical requirement factors, where "+" represents that the two technical requirements are positively correlated, "-" represents that the two technical requirements are negatively correlated, and "blank" represents that the two technical requirements have no correlation. means no correlation between the two technical requirements.

The degree of correlation between consumer demand and technical requirements is compared in the "room" of the quality house and is represented by a symbol. The correlation scores and symbols are shown in Table 11, from which the product characteristic scores are calculated.

**Table 11:** Correlation symbol.

Degree of Association	Strong	Medium	Weak	No association	Negative association
Symbol	◎	○	△	BLANKS	▽
Score	9	3	1	0	

**Fig 9:**
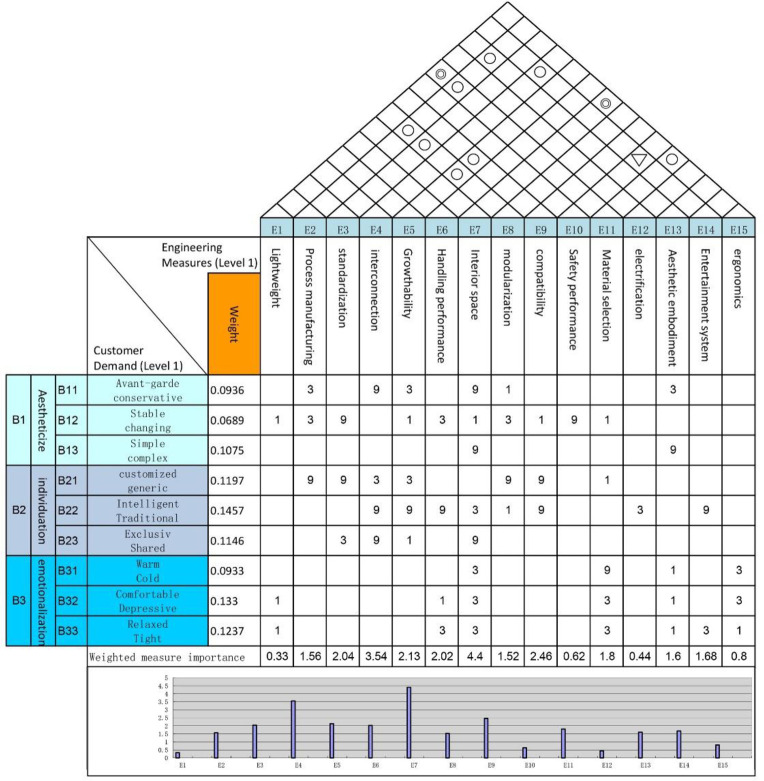
Comprehensive quality house.

The construction of the Integrated Quality House ensures a more accurate and efficient product design and development process by comprehensively analysing user needs and technical requirements, and comparing the degree of correlation between user needs and technical requirements, which allows the importance and priority of product features to be determined. The integrated quality house is shown in Fig 9.

## 4. Planning practice and discussion

### 4.1 Interconnection of interior space

Through the QFD of the interior space of the vehicle, it can be seen that vehicle connectivity is the key to growable design. The automotive design process takes into account the different stages of the full life cycle of the vehicle and the changing needs, and automotive connectivity provides a more comfortable and personalised experience for the user through the creative creation, development and enhancement of docking services, thus giving the vehicle a sustainable character [27]. Vehicle connectivity can take different forms and can be categorised into seven types, V2V (vehicle-to-vehicle communication), V2I (vehicle-to-infrastructure communication), V2X (vehicle-to-everything communication), V2P (vehicle-to-pedestrian communication), V2N (vehicle-to-network communication), V2D (vehicle-to-equipment communication), and V2C (vehicle-to-Cloud communication).The application of V2X communication technology can improve traffic safety, for example reducing driver-caused accidents by ensuring safe speeds, safe distances and safe driving styles.V2X communication technologies can be divided into three phases: improving driver information, improving traffic efficiency and safety, and solving complex driving situations [28].

The Internet of Vehicles (IoV) is the cornerstone of vehicle connectivity and aims to improve Intelligent Transportation System (ITS) by integrating data collection, data exchange, data computation and processing [29]. The rapid development of IoV field has triggered a focus on Human Vehicle Interaction (HVI). Intelligent interior spaces are being explored in order to improve user experience, acceptance and trust. More and more sensor networks are being integrated into vehicles to enable multimodal interactions, and Intelligent Vehicle Assistants (IVAs) equipped with large screen displays through immersive technologies such as Augmented Reality (AR) and Virtual Reality (VR) [30].

**Fig 10:**
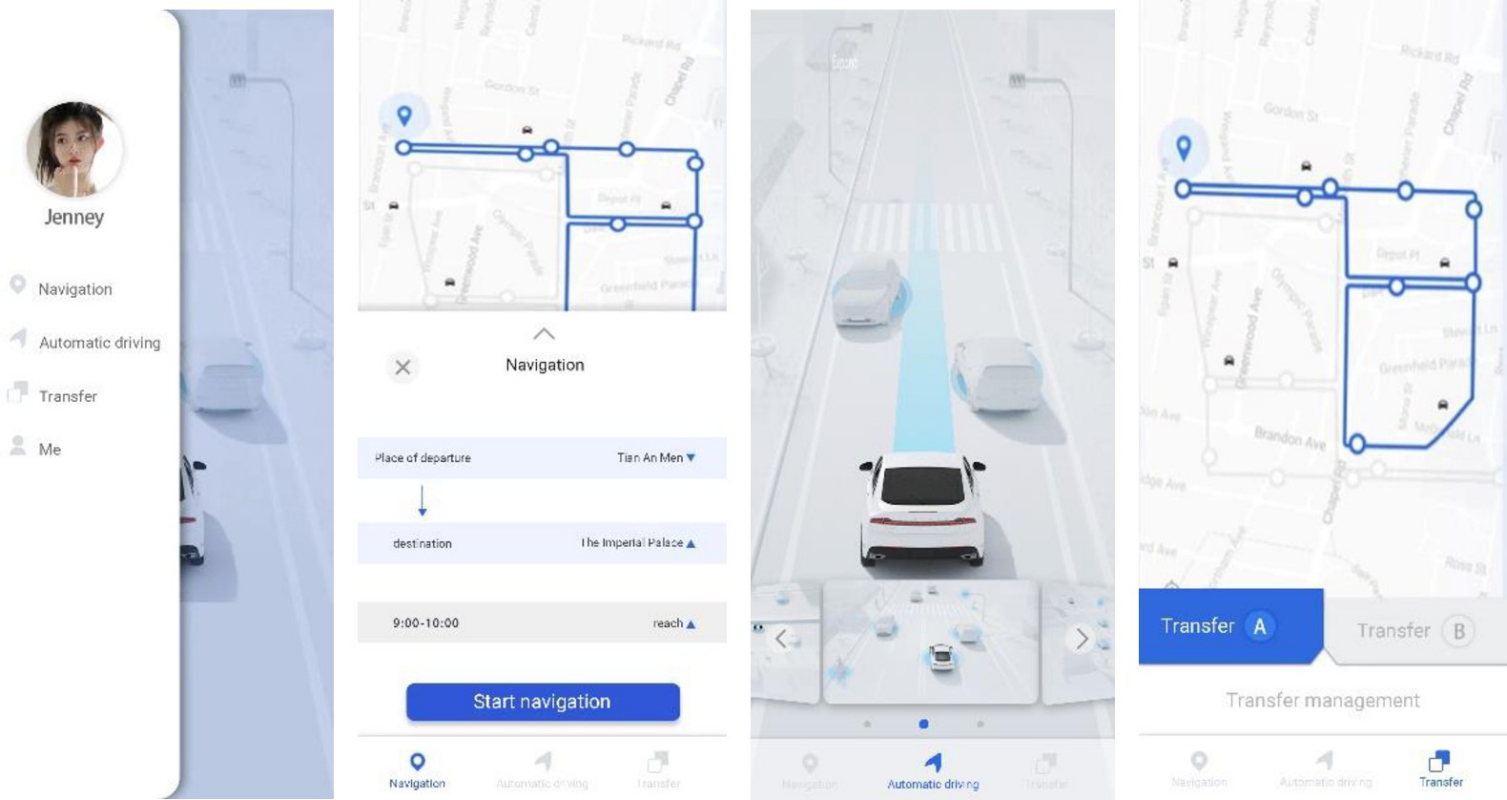
Mobile phone terminal interface.

As automotive technology continues to develop and evolve, the application of growable design embeds software interfaces in vehicles in order to integrate new technologies without altering them. For example, Tesla’s OTA technology can improve the software technology through the user’s feedback to improve the vehicle driving performance, and can also cover the mobile phone mobile terminal and travel service terminal to achieve a holistic travel service upgrade. Immersive virtual reality interaction technology works in the vehicle’s internal space environment, and the applicability and development of new user interface technologies can create more immersive experiences. From the behavioural, emotional and cognitive levels to participate in the digital system interaction, so the new experience of "immersive" vehicle environment was realised. Multi-modal interaction is the key to a vehicle’s interactive interface. Voice control allows passengers to interact with the vehicle without any physical contact, and the intelligent touch system automatically adjusts the number and level of supported functions according to the vehicle’s driving mode. In the design, the user’s mobile phone APP terminal needs to have navigation, autopilot, and handover takeover functions, as shown in Fig 10. In a highly autonomous driving state, the system will display more comfort, infotainment, and communication functions.

**Fig 11:**
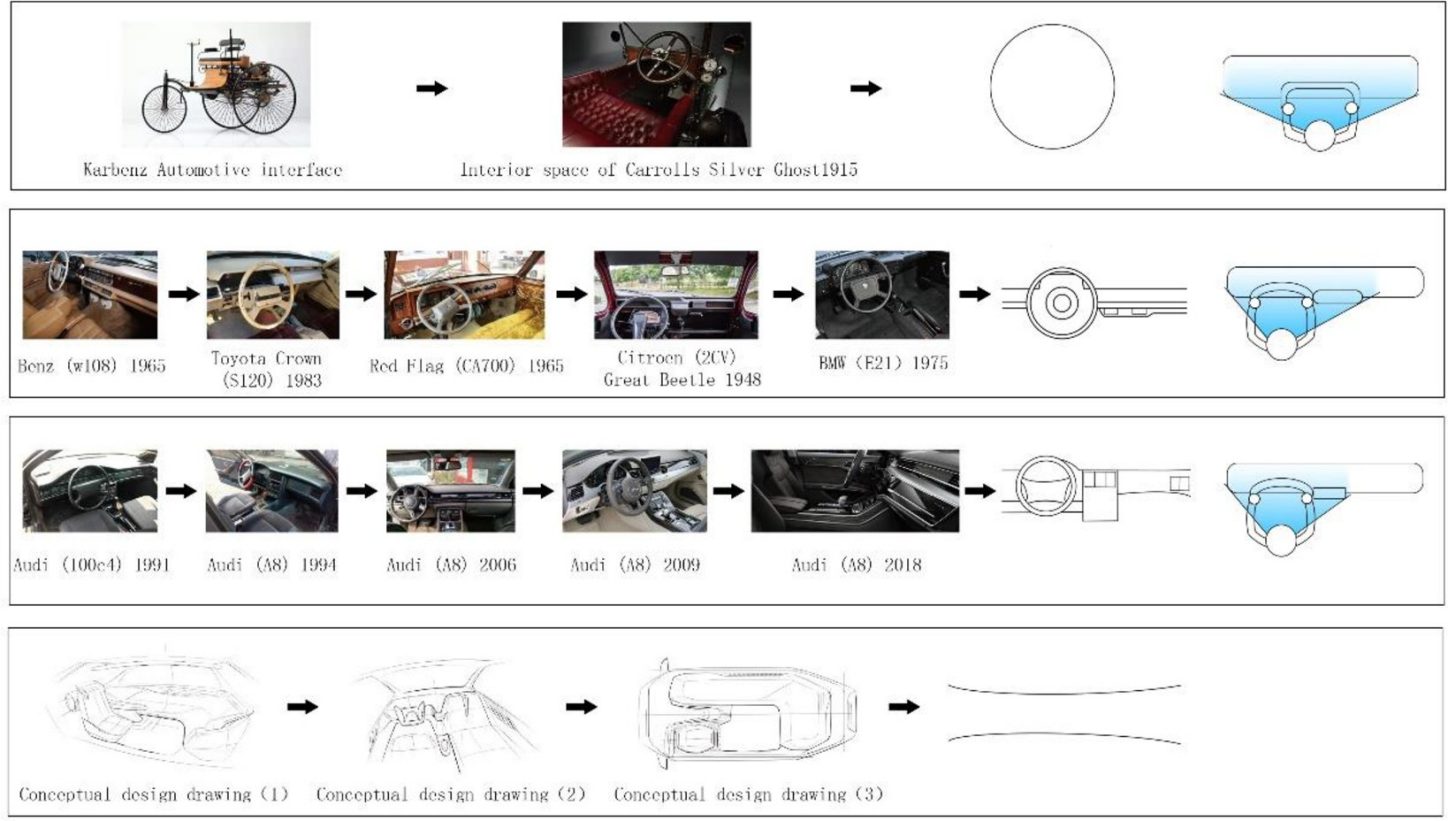
Automotive instrumentation design changes.

Through the extraction of the development law of the dashboard dynamic line, as shown in Fig 11. automotive instrument design change shows that with the development of the internal control interface, the multi-channel interaction mode has been formed, and the automotive instrument is gradually developing in the direction of screen-based and intelligent. Accompanied by the user’s needs for more functionality, the distribution of visual line of sight has gradually become smaller and tends to be centralised, which is conducive to improving the user’s efficiency. However, from the point of view of task operation, there are multi-task simultaneous operation, as well as round-trip switching mode between tasks, which has a clearer purposeful behaviour.

Hick’s law states that when designing an interactive interface, the distribution of interface information should be appropriate. This is because the driver can visually perceive between 5 and 9 items (7 ± 2) at a time. If it exceeds 9, it will put more cognitive load on the driver. The interactive interface should display environment, vehicle, and social media. Environment includes road conditions, weather, traffic signs, pedestrians, animals, etc.; vehicle aspect includes speedometer, technical gauge, fuel tank, warning signals, entertainment, etc.; social media includes streaming media, location information, entertainment, etc.

**Fig 12:**
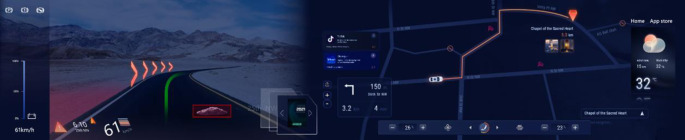
Vehicle interior dashboards.

This vehicle interior dashboard design weakens the centralisation of the driver’s seat, with the navigation and entertainment interfaces mainly occupying the middle position, allowing the vehicle passengers to view the progress from all angles, as shown in Fig 12. Entertainment functions and interactive modules are added to the information content, with the speed and RPM dashboards as the first tier, the map navigation interface as the second, and secondary information such as time and temperature as the third tier. It is also possible to break the inherent layout and personalise it according to the user’s preference and frequency of use. What’s more, it employs induction entry unlike traditional vehicle unlocking methods The facial recognition camera embedded in the B-pillar recognises the driver’s information and opens the door. The vehicle adopts hologram technology, the driver can control the interior air-conditioning and entertainment playback system with the touch of a fingertip, and the highly innovative 3D operation interface allows the driver to be fully immersed in the perfect experience of human-computer interaction.

### 4.2 Spacel layout of interior space

**Fig 13:**
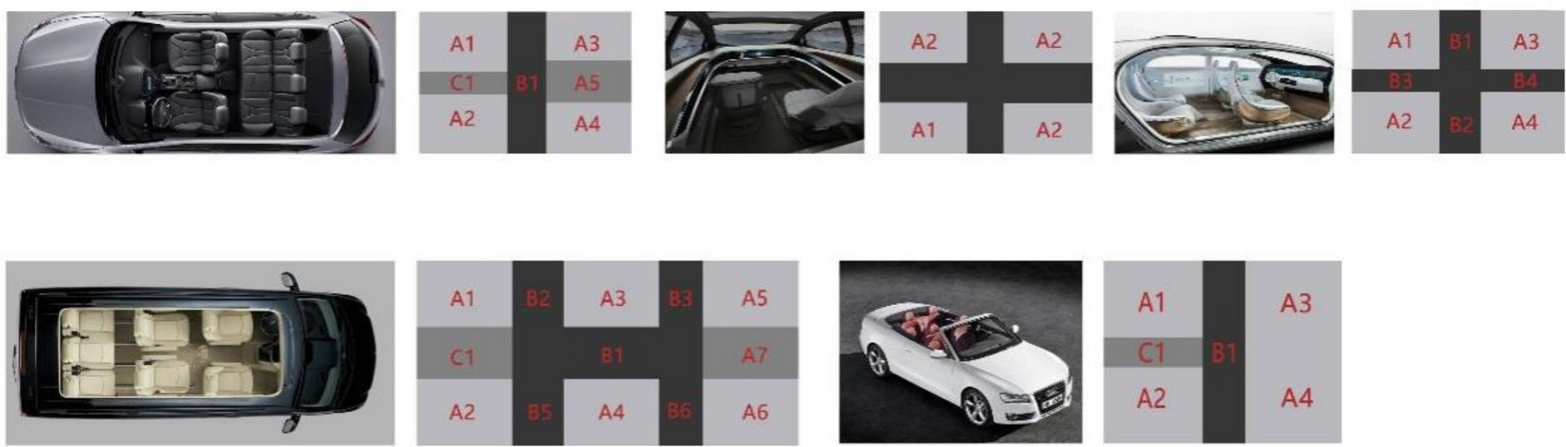
Conventional passenger vehicle interior space distribution layout.

Through QFD, it can be seen that the space layout is the most important in the growing design of the vehicle. With the continuous change of modern space requirements, people start to pursue multi-functional and diversified integrated passenger vehicle body space, which lead to that the original clear-cut interior space layout is gradually broken, each space is no longer a single existence, the boundaries of the space also gradually tend to blur, a space is given multiple functionality. By blending multiple functions into one space and by blurring the boundaries between different spaces, the space is made into a multifunctional area that adapts to different changes. Bill Hillier in the study of architectural space issues in the process of tangible material form, and even language and other immaterial forms, when we look at them as a relational system, we will find the existence of its "Configuration". Configuration is not confined to architecture, but seems to run through all fields that use a system of laws and function in a social way. The design point of the passenger vehicle seat in the passenger vehicle interior space design activity is crucial. Seat range space is the main activity space for drivers and passengers, and the distribution of seats in the interior space of passenger vehicles directly reflects people’s psychological perception of the privacy and openness of the interior space of passenger vehicles, space utilisation, comfort and enjoyment, as well as circulation and obstruction. The distribution of seats reflects the relationship between people and space as well as between people in passenger vehicle interiors. Obviously, the number of seats in a passenger vehicle is different, and the circulation of people in the interior space is different. In the interior space of a passenger vehicle, the range of human activity is mainly centred on the seats. Seats are analogous to "rooms" in architectural space, and the circulation is mainly the access space in the vicinity of the seats. Each seat in the interior of a passenger vehicle is represented by a hollow square, and the connection of the squares is the relationship between the seats, as shown in Fig 13. A: distribution of seat layout, B: distribution of aisle area, and C: distribution of operation and control attributes. Functional models with different functional attributes possess different space area distributions. Vehicles with different functional attributes have a different distribution of space areas.

Considering that the versatility of the interior space and the individual needs of the user are also contents of growth design, automotive modular platform has been developing rapidly in recent years. Automotive modular platform refers to the modular design and assembly of the various molecular systems of an automobile, and the various parts of the automobile assembly are standardised in the form of modules, and then finally "assembled" according to the positioning of different models. At present, including Fiat, Mercedes-Benz, Rinspeed, Rivian, Schaeffler and other traditional vehicle companies and emerging technology companies have launched a concept vehicle with interchangeable body. Volvo Vehicles has adopted a modular design in its Scalable Product Architecture, which allows it to share technology and components between different models.

Under the influence of ’function determines form’, the interior space of today’s passenger vehicles hasn’t changed much in terms of volume, but there have been developments in the styling of its components which the design-engineering match is becoming more and more demanding, and the design-led model is becoming more and more visible. As the engineering aspects become more stable and the styling aspects of design become more detailed, the study of the styling features of passenger vehicle interiors becomes more and more important. The study of feature surfaces is still based on feature lines, and the information obtained from the study of feature surfaces in isolation is similar to that of feature lines, so it is possible to adopt the research method of area units. For example, the internal space area is divided by seats or functional areas.

**Fig 14:**
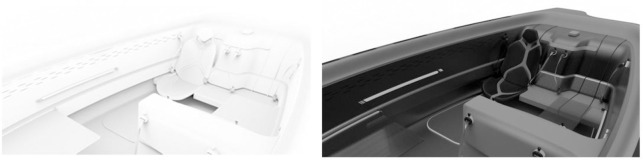
Integration of dashboard and door trims.

However, when considering other factors, such as better interaction needs required by the user, environmental needs in addition to the large number of horizontal elements should be considered, the whole vehicle should pay more attention to the integrated design of the dashboard and door trim, a large number of three-dimensional section treatment is conducive to strengthen the passenger vehicle interior space area vision, as shown in Fig 14.

Traditional vehicle interior space is relatively rigid that the vehicle interior area can generally be divided into three functional areas. the first level area is usually the owner and co-pilot activity area, the front seats and the dashboard; the second level area is generally the passenger area, like rear seats; the third level area is the storage area, like the boot. Specifically, the first level area is used frequently, with more space, no moving area, and a small expansion area. The principle of storage is to make it easy to access, which is mainly for the driver. also, Items that are stored in the first level area are used more frequently, Such as mobile phones, high-speed toll vehicled, sunglasses, cash and so on. The space in the second level area of is smaller so that the movable area and the expansion area are small, which is generally designed in the seat back and door panels. The third level area is the boot of the vehicle, mainly for storage purposes, part of the three-box vehicle rear seats can be put down to increase the storage space when needed.

**Fig 15:**
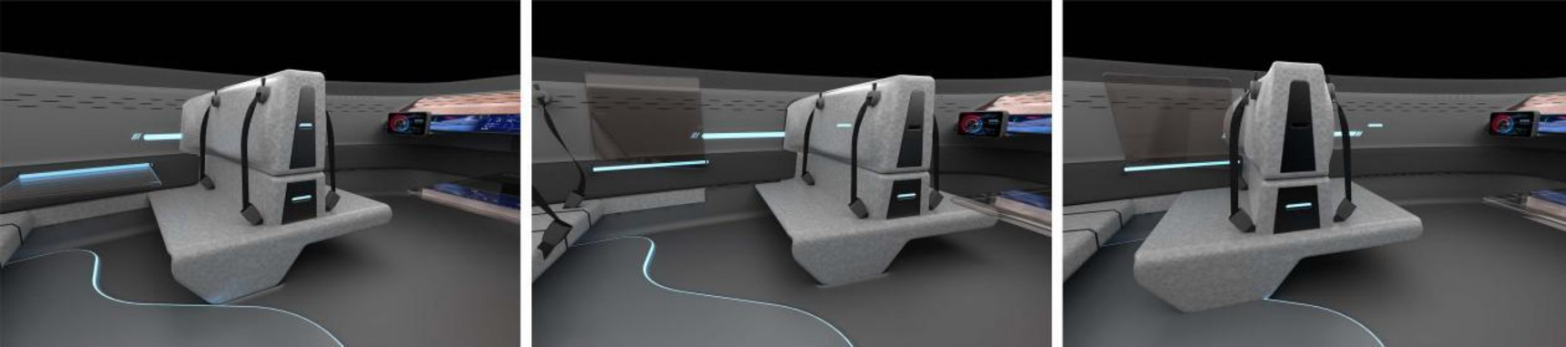
Mobility of space.

The L5 level of autopilot makes the driver’s compartment progressively less functional and will become an emergency seat when extra passengers appear. The interior of this vehicle has an oversized horizontal dashboard running to the doors with eliminating the traditional engine compartment and adopting a living room layout. Moreover, the seat moves flexibly in the vehicle and rotates in all directions. Intelligent seat is currently defined as an in-vehicle seat with certain human brain-like intelligence, and its working principle is that the signal is sent from the pressure sensor, which is accepted by the central control system and acted on the worm gear drive mechanism, and at the same time detects the signals accepted by the various pressure sensors on the seat, which are fed back to the central control system and vehiclery out certain simple operations. It can change different arrangements in different scenarios to achieve spatial mobility, and can vehiclery more scenarios experience to adapt to the different needs of passengers, as in Fig 15. The steering wheel is also designed to be foldable and hidden, together with the driver-centred ring-type retina screen, which can bring a multi-dimensional and integrated reality interaction experience. The foldable table at the inner door panel can be folded and opened according to the needs of the occupants to realise space expansion.

### 4.3 Perceived experience of interior space

Users’ needs for automobiles have changed from satisfying transport needs to emotional satisfaction with the interior space of the vehicle, which means that drivers and passengers recognise the mental and physical space of the vehicle. Due to cost and physical constraints, it is difficult for vehicle companies to expand the physical space of the vehicle, so they tend to work on expanding the perceived space [31]. Psychological interaction needs depend on intelligent emotional interaction and physical mediated interaction. The role played by vehicles in the life of urban residents has long jumped out of the realm of mobility tools. More often, the vehicle will be used as office space, entertainment space and even living space, so it is necessary to pay more attention to the "emotional" design. on one hand, it is the "human touch" interaction experience, on the other hand, is to reflect the vehicle of the hardware facilities.

**Fig 16:**
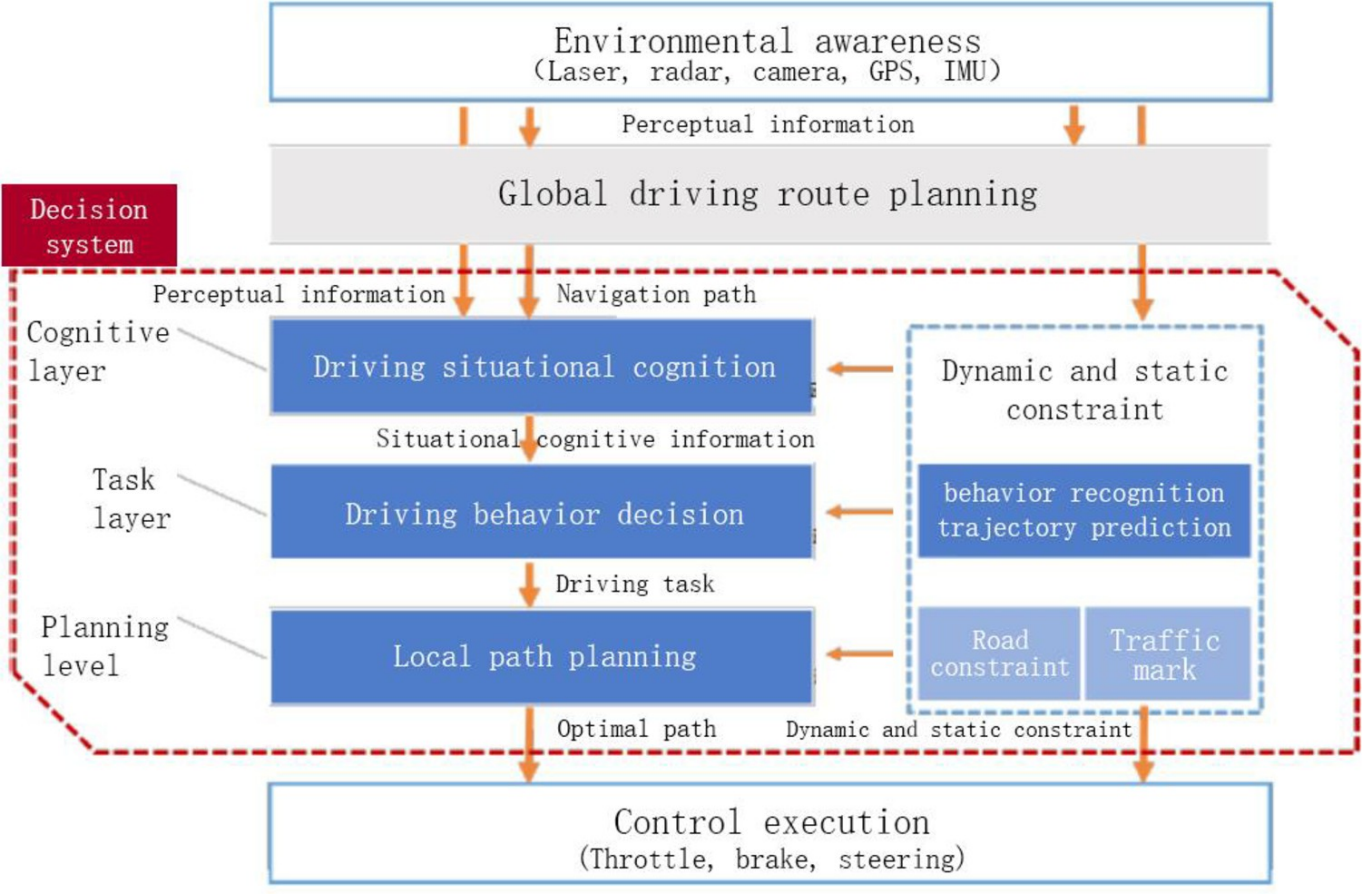
Driver cognition system.

To assess the user’s psychological interaction needs, we need to obtain the driver’s cognitive system from the consumer’s cognition, task layer and planning layer based on the principles of cognitive psychology, which describes the driver’s process of information processing in the driving process. Human as a decision maker in going through the stage of perceiving information to the stage of judgement decision-making, and finally the stage of reaction, as shown in Fig 16.

**Fig 17:**
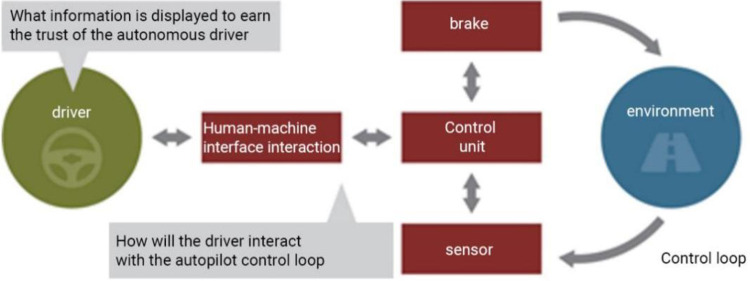
Security mechanism.

As human trust in AI is still in its infancy, the market needs a certain transition period for consumers to complete this psychological transformation. This concept vehicle is equipped with a driverless system of L5, where the steering wheel and pedals can be activated when needed. The relationship between the driver and the automated driving system for receiving information is something that needs to be further investigated, A.T. Kearney [32] analysed that the driver should establish a safety mechanism with the system to improve risk perception and trust as shown in Fig 17. The human-computer interface is the key to win the trust of automated driving. The control unit contains brakes and sensors that collect information about the external environment and feed it back to the sensors through a control loop to form a closed loop.

**Fig 18:**
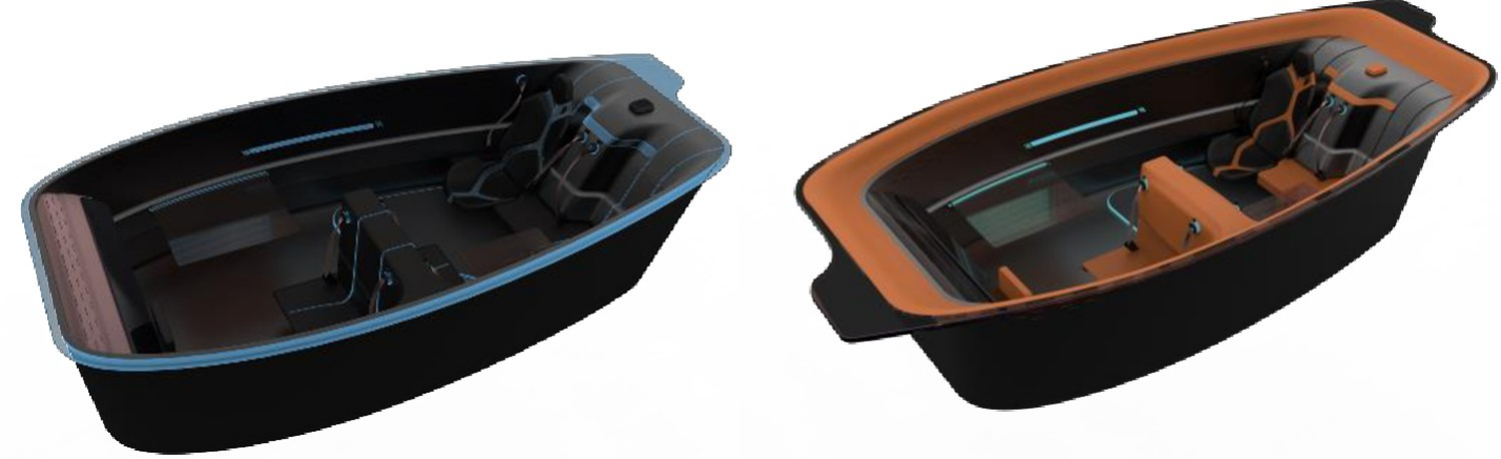
Interior space colour scheme.

Human needs are diverse and can be broadly divided into two elements, physical needs and spiritual needs, for different needs colours and interior styles will be produced in difference. Physiological needs and safety needs correspond to the material needs of people, which are expressed in the colour of the vehicle interior as the functionality of the colour embodied in the interior, affecting the usability of the product. Colours in the main driving area should be chosen to facilitate driving and reduce driver fatigue, which requires that the colours should not be too stimulating and highly reflective; while in the passenger area, colours should be chosen to make the user feel calm, comfortable and conducive to their rest. In terms of overall space, lighter interior colours will appear more spacious than darker ones. By considering the user’s needs, two colour schemes were designed, as shown in Fig 18.

## 5. Conclusions

In summary, with the shortening of the research and development cycle of intelligent vehicles, automotive products have undergone a qualitative transformation. A growable automotive interior space design can effectively satisfy users’ personalised customisation preferences, enhance users’ purchase intention, and mitigate environmental pollution problems. In this study, the FAHP method was used to comprehensively analyse and rank the SET series of factors, combined with the grey correlation method to correlate with the areas related to the interior space of the vehicle, and constructed a sample of the interior space of the vehicle. By extracting the kansei words of the spatial sample, the average score of the intended vocabulary was calculated by applying the kansei engineering method, and factor analysis was performed. Next, a fuzzy QFD quality house was established to correlate the technical requirements with the perceptual factors and derive the design weights, which guide to design. This study illustrates the design methodology of automotive interior space from three perspectives of interior space connectivity, spatial layout and perceptual experience to achieve automotive interior space programme design. The results of the study provide systematic guidance for growable automotive interaction design and valuable suggestions for subsequent interior development. However, this study needs further exploration of the ergonomic and materialistic aspects of automobiles’ interior space. Therefore, future research should focus on ergonomic influences such as automotive seating, driver visual perception, and sustainability of seating and interior materials.

## Supporting information

S1 Dataset (ZIP)
