# Supplementary material for: Growable design of passenger vehicle interior space based on FAHP and FQFD
Source: PLoS One. 2024 Jun 20;19(6):e0303233. doi: 10.1371/journal.pone.0303233 (PMC11189197; doi:10.1371/journal.pone.0303233)
Supplement: S1 Dataset — (ZIP) [file pone.0303233.s001.zip › Data sets used in the paper/Distance to clustering center.rtf]

Cluster Members	
Case Number	V1	Collection	Distance	
1	A1	1	20.790	
2	A2	1	14.591	
3	A3	1	15.521	
4	A4	4	19.155	
5	A5	2	24.339	
6	A6	3	21.743	
7	A7	2	22.285	
8	A8	3	21.435	
9	A9	2	24.130	
10	A10	4	20.265	
11	A11	1	18.015	
12	A12	3	19.080	
13	A13	2	23.536	
14	A14	1	16.028	
15	A15	4	21.608	
16	A16	4	24.823	
17	A17	4	27.091	
18	A18	4	23.694	
19	A19	2	22.441	
20	A20	3	18.045	
21	A21	1	21.429	
22	A22	3	22.917	
23	A23	3	18.786	
24	A24	2	19.421	
25	A25	4	19.369	
26	A26	2	19.071	
27	A27	3	23.016	
28	A28	4	23.461	
29	A29	2	22.510	
30	A30	2	24.066	

Number of observations in each cluster	
Collection	1	6.000	
	2	9.000	
	3	7.000	
	4	8.000	
Efficiently	30.000	
Omissions	.000	
