# Supplementary material for: Growable design of passenger vehicle interior space based on FAHP and FQFD
Source: PLoS One. 2024 Jun 20;19(6):e0303233. doi: 10.1371/journal.pone.0303233 (PMC11189197; doi:10.1371/journal.pone.0303233)
Supplement: S1 Dataset — (ZIP) [file pone.0303233.s001.zip › Data sets used in the paper/Factor analysis data.docx]

**Factor analysis**

| **附註** | | |
| --- | --- | --- |
| Output established | | 09-OCT-2023 14:01:12 |
| Remarks | |  |
| Input | Data sets in action | Data set 1 |
|  | Filters | <No> |
|  | Coarse and fine | <No> |
|  | Split file | <No> |
|  | Column N in the job data file | 36 |
| Missing Value Handling | Definition of Omission | MISSING=EXCLUDE: User-defined missing values are considered missing. |
|  | Using Observations | LISTWISE: The statistics are based on observations with no missing values for any of the variables used. |
| Grammar | | FACTOR  /VARIABLES @2 Avant-garde Conservative @3 Advanced Backward @4 Warm Cold @7 Stable Changing @10 Soothing Depressing @12 Customised Generic @14 Intelligent Traditional @20 Exclusive Shared @23 Practical Wasteful @24 Minimalist Complicated @25 Relaxing Tight @26 Simple Complicated  /MISSING LISTWISE  /ANALYSIS @2 Avant-garde Conservative @3 Advanced Backward @4 Warm Cold @7 Stable Changing @10 Soothing Depressing @12 Customised Generic @14 Intelligent Conventional @20 Exclusive Shared @23 Utilitarian Wasteful @24 Simple Exuberant @25 Effortless Taut @26 Succinctly Complex  /PRINT INITIAL CORRELATION KMO EXTRACTION ROTATION FSCORE  /PLOT EIGEN ROTATION  /CRITERIA MINEIGEN(1) ITERATE(25)  /EXTRACTION PC  /CRITERIA ITERATE(25) /EXTRACTION PC  /ROTATION VARIMAX  /SAVE REG(ALL)  /METHOD=CORRELATION. |
| Resources | Processor Time | 00:00:00.06 |
|  | Experience Time | 00:00:00.36 |
|  | Maximum Memory Required | 20256 (19.781K) Bytes |
| Variables created | FAC1_1 | Component Rating 1 |
|  | FAC2_1 | Component Rating 2 |
|  | FAC3_1 | Component Rating 3 |

**[Dataset 1]**

| **Correlation matrix** | | | | | |
| --- | --- | --- | --- | --- | --- |
|  | | 2 Avant-garde - conservative | 3 Advanced - Backward | 4 Warm - Cold | 7 Stable - changing |
| Related | 2 Avant-garde - conservative | 1.000 | .130 | -.017 | -.370 |
|  | 3 Advanced - Backward | .130 | 1.000 | -.247 | .076 |
|  | 4 Warm - Cold | -.017 | -.247 | 1.000 | -.161 |
|  | 7Stable - Changing | -.370 | .076 | -.161 | 1.000 |
|  | 10 Soothing - Depressing | .042 | -.054 | .421 | -.281 |
|  | 12Customised - Generic | .210 | .446 | -.045 | -.337 |
|  | 14 Intelligent - Traditional | -.163 | .389 | -.209 | -.084 |
|  | 20 Exclusive - Shared | -.180 | -.338 | -.062 | .284 |
|  | 23Practical - Wasteful | -.200 | -.162 | .464 | .202 |
|  | 24 Simple - Complicated | .546 | .195 | -.238 | -.404 |
|  | 25 Relaxed - Tight | -.003 | -.035 | .427 | -.391 |
|  | 26 Simple - Complex | .540 | .188 | -.289 | -.406 |

| **Correlation matrix** | | | | | |
| --- | --- | --- | --- | --- | --- |
|  | | 10 Soothing - Depressing | 12 Customised - Generic | 14 Intelligent - Traditional | 20 Exclusive - Shared |
| Related | 2 Avant-garde - conservative | .042 | .210 | -.163 | -.180 |
|  | 3 Advanced - Backward | -.054 | .446 | .389 | -.338 |
|  | 4 Warm - Cold | .421 | -.045 | -.209 | -.062 |
|  | 7Stable - Changing | -.281 | -.337 | -.084 | .284 |
|  | 10 Soothing - Depressing | 1.000 | .328 | .100 | -.387 |
|  | 12Customised - Generic | .328 | 1.000 | .777 | -.667 |
|  | 14 Intelligent - Traditional | .100 | .777 | 1.000 | -.465 |
|  | 20 Exclusive - Shared | -.387 | -.667 | -.465 | 1.000 |
|  | 23Practical - Wasteful | .119 | -.209 | -.102 | .030 |
|  | 24 Simple - Complicated | .089 | .437 | .213 | -.231 |
|  | 25 Relaxed - Tight | .615 | .169 | -.038 | .035 |
|  | 26 Simple - Complex | .075 | .447 | .241 | -.242 |

| **Correlation matrix** | | | | | |
| --- | --- | --- | --- | --- | --- |
|  | | 23 Practical - Wasteful | 24 Simple - Complex | 25 Relaxed - Tight | 26 Simple - Complex |
| Related | 2 Avant-garde - conservative | -.200 | .546 | -.003 | .540 |
|  | 3 Advanced - Backward | -.162 | .195 | -.035 | .188 |
|  | 4 Warm - Cold | .464 | -.238 | .427 | -.289 |
|  | 7Stable - Changing | .202 | -.404 | -.391 | -.406 |
|  | 10 Soothing - Depressing | .119 | .089 | .615 | .075 |
|  | 12Customised - Generic | -.209 | .437 | .169 | .447 |
|  | 14 Intelligent - Traditional | -.102 | .213 | -.038 | .241 |
|  | 20 Exclusive - Shared | .030 | -.231 | .035 | -.242 |
|  | 23Practical - Wasteful | 1.000 | -.254 | .022 | -.288 |
|  | 24 Simple - Complicated | -.254 | 1.000 | .135 | .984 |
|  | 25 Relaxed - Tight | .022 | .135 | 1.000 | .134 |
|  | 26 Simple - Complex | -.288 | .984 | .134 | 1.000 |

| **KMO and Bartlett Testing** | | |
| --- | --- | --- |
| Kaiser-Meyer-Olkin Measurement of sampling appropriateness. | | .566 |
| Bartlett's Spherical Determination | Approximate Cardinality | 274.748 |
|  | DF | 66 |
|  | Significance | .000 |

| **Communalities** | | |
| --- | --- | --- |
|  | Originate | Retrieve |
| 2 Avant-garde - conservative | 1.000 | .570 |
| 3 Advanced - Backward | 1.000 | .461 |
| 4 Warm - Cold | 1.000 | .711 |
| 7Stable - Changing | 1.000 | .563 |
| 10 Soothing - Depressing | 1.000 | .713 |
| 12Customised - Generic | 1.000 | .886 |
| 14 Intelligent - Traditional | 1.000 | .776 |
| 20 Exclusive - Shared | 1.000 | .625 |
| 23Practical - Wasteful | 1.000 | .347 |
| 24 Simple - Complicated | 1.000 | .840 |
| 25 Relaxed - Tight | 1.000 | .611 |
| 26 Simple - Complex | 1.000 | .859 |

| **Retrieval method: main component analysis.** |
| --- |

| **Total number of variables stated** | | | | | | | | |
| --- | --- | --- | --- | --- | --- | --- | --- | --- |
| Element | Starting Characteristics | | | Capture Square and Load | | | Load Cyclic Sum of Squares | |
|  | Total | Variable % | Accumulation % | Total | Variable % | Accumulation % | Total | Variable % |
| 1 | 3.756 | 31.304 | 31.304 | 3.756 | 31.304 | 31.304 | 2.917 | 24.305 |
| 2 | 2.318 | 19.316 | 50.620 | 2.318 | 19.316 | 50.620 | 2.698 | 22.480 |
| 3 | 1.888 | 15.734 | 66.354 | 1.888 | 15.734 | 66.354 | 2.348 | 19.569 |
| 4 | .986 | 8.219 | 74.573 |  |  |  |  |  |
| 5 | .790 | 6.582 | 81.155 |  |  |  |  |  |
| 6 | .762 | 6.350 | 87.505 |  |  |  |  |  |
| 7 | .551 | 4.591 | 92.096 |  |  |  |  |  |
| 8 | .389 | 3.239 | 95.336 |  |  |  |  |  |
| 9 | .292 | 2.435 | 97.770 |  |  |  |  |  |
| 10 | .163 | 1.361 | 99.131 |  |  |  |  |  |
| 11 | .092 | .769 | 99.900 |  |  |  |  |  |
| 12 | .012 | .100 | 100.000 |  |  |  |  |  |

| **Total number of variables stated** | |
| --- | --- |
| Element | Load Cyclic Sum of Squares |
|  | Accumulation % |
| 1 | 24.305 |
| 2 | 46.785 |
| 3 | 66.354 |
| 4 |  |
| 5 |  |
| 6 |  |
| 7 |  |
| 8 |  |
| 9 |  |
| 10 |  |
| 11 |  |
| 12 |  |

| **Retrieval method: main component analysis.** |
| --- |


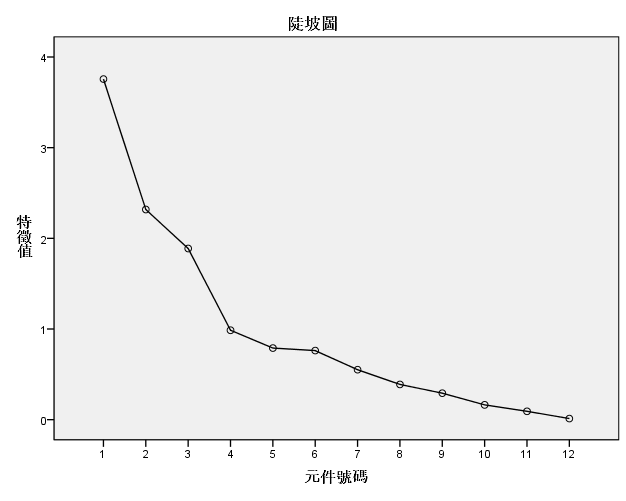


| **Component matrixa** | | | |
| --- | --- | --- | --- |
|  | Element | | |
|  | 1 | 2 | 3 |
| 2 Avant-garde - conservative | .516 | -.056 | -.548 |
| 3 Advanced - Backward | .437 | -.294 | .428 |
| 4 Warm - Cold | -.185 | .823 | .003 |
| 7Stable - Changing | -.556 | -.372 | .341 |
| 10 Soothing - Depressing | .315 | .774 | .118 |
| 12Customised - Generic | .824 | .086 | .446 |
| 14 Intelligent - Traditional | .554 | -.125 | .674 |
| 20 Exclusive - Shared | -.617 | -.175 | -.462 |
| 23Practical - Wasteful | -.368 | .418 | .192 |
| 24 Simple - Complicated | .801 | -.147 | -.420 |
| 25 Relaxed - Tight | .225 | .730 | -.165 |
| 26 Simple - Complex | .813 | -.175 | -.409 |

| Retrieval method: main component analysis. a |
| --- |
| a. Retrieve 3 components. |

| **Rotating element matrixa** | | | |
| --- | --- | --- | --- |
|  | Element | | |
|  | 1 | 2 | 3 |
| 2 Avant-garde - conservative | .750 | -.069 | .050 |
| 3 Advanced - Backward | .084 | .627 | -.247 |
| 4 Warm - Cold | -.258 | -.168 | .785 |
| 7Stable - Changing | -.581 | -.087 | -.467 |
| 10 Soothing - Depressing | .042 | .250 | .805 |
| 12Customised - Generic | .303 | .872 | .183 |
| 14 Intelligent - Traditional | -.016 | .877 | -.077 |
| 20 Exclusive - Shared | -.127 | -.742 | -.241 |
| 23Practical - Wasteful | -.459 | -.123 | .349 |
| 24 Simple - Complicated | .890 | .220 | -.005 |
| 25 Relaxed - Tight | .169 | -.019 | .763 |
| 26 Simple - Complex | .895 | .237 | -.032 |

| Retrieval method: main component analysis.  Axis method: maximum variation method with Kaiser normalisation. a |
| --- |
| a. Converge the cycle in 4 overlapping generations. |

| **Component Conversion Matrix** | | | |
| --- | --- | --- | --- |
| Element | 1 | 2 | 3 |
| 1 | .739 | .658 | .148 |
| 2 | -.145 | -.059 | .988 |
| 3 | -.658 | .751 | -.052 |

| Retrieval method: Principal component analysis.  Rotation method: Maximum variation method with Kaiser normalisation. |
| --- |


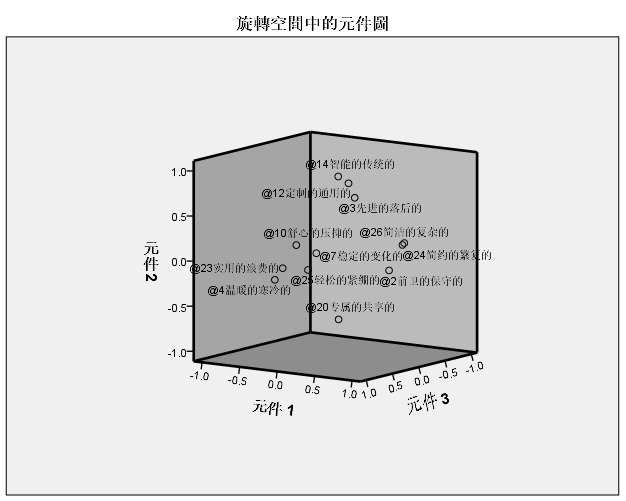


| **Component Scoring Coefficient Matrix** | | | |
| --- | --- | --- | --- |
|  | Element | | |
|  | 1 | 2 | 3 |
| 2 Avant-garde - conservative | .296 | -.126 | .012 |
| 3 Advanced - Backward | -.045 | .254 | -.120 |
| 4 Warm - Cold | -.089 | -.052 | .343 |
| 7Stable - Changing | -.205 | .048 | -.189 |
| 10 Soothing - Depressing | -.028 | .082 | .339 |
| 12Customised - Generic | .001 | .320 | .057 |
| 14 Intelligent - Traditional | -.118 | .368 | -.050 |
| 20 Exclusive - Shared | .051 | -.287 | -.086 |
| 23Practical - Wasteful | -.165 | .001 | .159 |
| 24 Simple - Complicated | .313 | -.023 | -.020 |
| 25 Relaxed - Tight | .056 | -.045 | .324 |
| 26 Simple - Complex | .314 | -.016 | -.032 |

| Retrieval method: Principal component analysis.  Rotation method: Maximum variation method with Kaiser normalisation.  Component Scoring. |
| --- |

| **Component Scoring Covariant Anomalous Matrix** | | | |
| --- | --- | --- | --- |
| Element | 1 | 2 | 3 |
| 1 | 1.000 | .000 | .000 |
| 2 | .000 | 1.000 | .000 |
| 3 | .000 | .000 | 1.000 |

| Retrieval method: Principal component analysis.  Rotation method: Maximum variation method with Kaiser normalisation.  Component Scoring. |
| --- |
