# Supplementary material for: Growable design of passenger vehicle interior space based on FAHP and FQFD
Source: PLoS One. 2024 Jun 20;19(6):e0303233. doi: 10.1371/journal.pone.0303233 (PMC11189197; doi:10.1371/journal.pone.0303233)
Supplement: S1 Dataset — (ZIP) [file pone.0303233.s001.zip › Data sets used in the paper/FAHP analysis data.docx]

Fuzzy judgement matrix A and weighting data

| A | SocialA1 | EconomicsA2 | TechnicalA3 | W |
| --- | --- | --- | --- | --- |
| SocialA1 | 0.5 | 0.65 | 0.55 | 0.3667 |
| EconomicsA2 | 0.35 | 0.5 | 0.4 | 0.2917 |
| TechnicalA3 | 0.45 | 0.6 | 0.5 | 0.3417 |

Fuzzy judgement matrix A1 and weighting data

| A1 | A11 | A12 | A13 | A14 | W |
| --- | --- | --- | --- | --- | --- |
| A11 | 0.5 | 0.4 | 0.55 | 0.65 | 0.2583 |
| A12 | 0.6 | 0.5 | 0.7 | 0.8 | 0.3000 |
| A13 | 0.45 | 0.3 | 0.5 | 0.6 | 0.2375 |
| A14 | 0.35 | 0.2 | 0.4 | 0.5 | 0.2042 |

Fuzzy judgement matrix A2 and weighting data

| A1 | A21 | A22 | A23 | A24 | W |
| --- | --- | --- | --- | --- | --- |
| A21 | 0.5 | 0.2 | 0.45 | 0.4 | 0.2125 |
| A22 | 0.8 | 0.5 | 0.6 | 0.55 | 0.2875 |
| A23 | 0.55 | 0.4 | 0.5 | 0.55 | 0.2500 |
| A24 | 0.6 | 0.45 | 0.45 | 0.5 | 0.2500 |

Fuzzy judgement matrix A3 and weighting data

| A1 | A31 | A32 | A33 | A34 | W |
| --- | --- | --- | --- | --- | --- |
| A31 | 0.5 | 0.6 | 0.55 | 0.3 | 0.2485 |
| A32 | 0.4 | 0.5 | 0.45 | 0.25 | 0.2167 |
| A33 | 0.45 | 0.55 | 0.5 | 0.4 | 0.2417 |
| A34 | 0.7 | 0.75 | 0.6 | 0.5 | 0.2958 |

Combined Weighting of SET Factors for Vehicle Interior Space

| Factor | *A11* | *A12* | *A13* | *A14* | *A21* | *A22* | *A23* | *A24* | *A31* | *A32* | *A33* | *A34* |
| --- | --- | --- | --- | --- | --- | --- | --- | --- | --- | --- | --- | --- |
| Combined Weight | 0.0947 | 0.11 | 0.087 | 0.0749 | 0.062 | 0.0839 | 0.0729 | 0.0729 | 0.084 | 0.074 | 0.0826 | 0.101 |
| Ranking | 3 | 1 | 4 | 8 | 12 | 6 | 10 | 10 | 5 | 9 | 7 | 2 |
